# A Critical Review of Data Science Applications in Resource Recovery and Carbon Capture from Organic Waste

**DOI:** 10.1021/acsestengg.3c00043

**Published:** 2023-09-29

**Authors:** Mohammed T. Zaki, Lewis S. Rowles, Donald A. Adjeroh, Kevin D. Orner

**Affiliations:** † Wadsworth Department of Civil and Environmental Engineering, West Virginia University, Morgantown, West Virginia 26505, United States; ‡ Department of Civil Engineering and Construction, Georgia Southern University, Statesboro, Georgia 30458, United States; § Lane Department of Computer Science and Electrical Engineering, West Virginia University, Morgantown, West Virginia 26505, United States

**Keywords:** Energy Recovery, Nutrient Management, Decarbonization, Machine Learning, Life Cycle Assessment

## Abstract

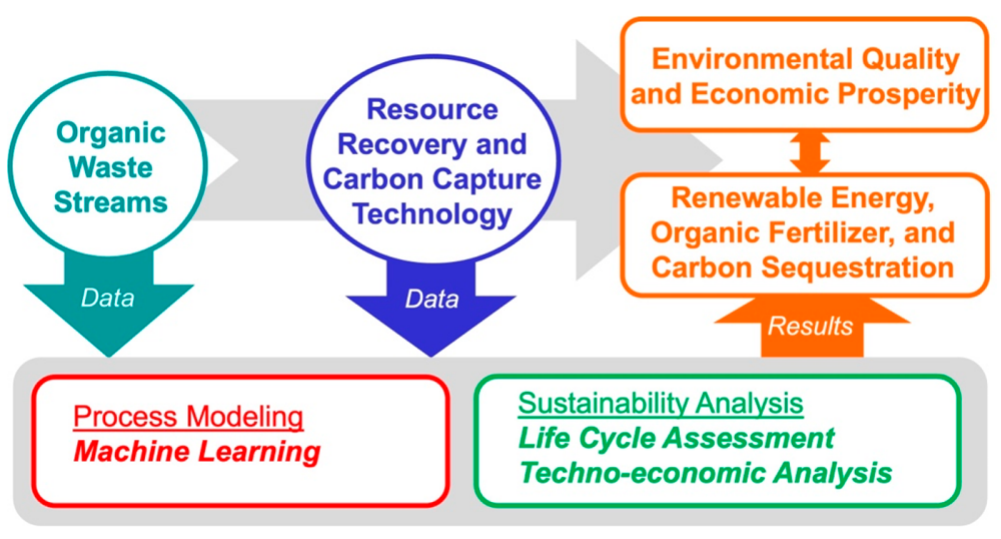

Municipal and agricultural organic waste can be treated to recover energy, nutrients, and carbon through resource recovery and carbon capture (RRCC) technologies such as anaerobic digestion, struvite precipitation, and pyrolysis. Data science could benefit such technologies by improving their efficiency through data-driven process modeling along with reducing environmental and economic burdens via life cycle assessment (LCA) and techno-economic analysis (TEA), respectively. We critically reviewed 616 peer-reviewed articles on the use of data science in RRCC published during 2002–2022. Although applications of machine learning (ML) methods have drastically increased over time for modeling RRCC technologies, the reviewed studies exhibited significant knowledge gaps at various model development stages. In terms of sustainability, an increasing number of studies included LCA with TEA to quantify both environmental and economic impacts of RRCC. Integration of ML methods with LCA and TEA has the potential to cost-effectively investigate the trade-off between efficiency and sustainability of RRCC, although the literature lacked such integration of techniques. Therefore, we propose an integrated data science framework to inform efficient and sustainable RRCC from organic waste based on the review. Overall, the findings from this review can inform practitioners about the effective utilization of various data science methods for real-world implementation of RRCC technologies.

## 1 Introduction

Improper organic waste management practices such as landfilling and incineration contribute to global warming through increased greenhouse gas emissions, cause environmental degradation due to runoff and leaching of contaminants, and degrade human health by spreading pathogens.^1−3^ Economic impacts of improper organic waste management include annual losses of USD 2.2 billion caused by eutrophication of freshwater bodies in the United States of America (USA) and USD 1 billion from food waste and food loss on a global scale.^4,5^ Although the Sustainable Development Goals (SDG) set by the United Nations motivate efforts to valorize organic waste through resource recovery and carbon capture (RRCC), limited progress has been made.^6−9^ For example, the 2021 SDG progress report estimated that globally 82% of municipal solid waste is collected, whereas only 55% is managed in controlled facilities (e.g., landfill site, incineration with energy recovery, composting).^10^ Distinct opportunities for RRCC from such waste exist to help address SDG target 12.5, which involves substantially reducing waste generation by 2030 through prevention, reduction, recycling, and reuse.^10^

Fortunately, these organic waste streams, rich in nutrients (e.g., nitrogen, phosphorus, potassium) and carbon, can be valorized utilizing RRCC technologies while also mitigating risks to the environment and human health.^2,11−15^ On a global scale, a fully functional circular economy between agriculture and wastewater treatment utilizing RRCC could have reduced 140 Tg of CO_2_ emissions in addition to sequestering 104 Tg of CO_2_ in 2022.^16^ RRCC from organic waste streams also addresses two of the 14 grand challenges for engineering in the 21st century: managing the nitrogen cycle and developing carbon sequestration methods.^17^ Laboratory-scale implementations of RRCC technologies are plentiful in the literature compared to their full-scale implementation.^18−22^ Scaling up such laboratory-scale technologies requires analyzing not only additional interconnected components but also the already existing operational complexity of RRCC such as impacts on the economy and the environment. Hence, exploiting opportunities to reduce costs and environmental impacts while improving their operational efficiency can be challenging but still attractive for decision-makers.

Data science has been used as an effective tool to improve the efficiency of RRCC technologies. Data science combines mathematical principles with process algorithms and can be applied to laboratory and/or field data in progressive stages to extract meaningful insights.^23−26^ These insights can help decision-makers make informed choices regarding the environmental and economic impacts of an RRCC technology. More specifically, applications of data science in RRCC from organic waste streams typically involve data-driven approaches for the modeling of treatment processes as well as methods for analyzing environmental and economic impacts of the technologies. Several studies over the past two decades have utilized data-driven approaches such as statistical techniques and machine learning (ML) methods to model processes of various RRCC technologies from different organic waste streams.^27−29^ More commonly, studies have focused on assessing the environmental and/or economic impacts of such technologies utilizing life cycle assessments (LCA) and life cycle cost analysis (LCCA)/techno-economic analysis (TEA).^30−32^ Limited studies have integrated statistical and ML methods with LCA and LCCA/TEA to evaluate the operational efficiency and environmental-economic impacts of RRCC technologies.^33−35^

Due to increasing accessibility of such data science tools (e.g., open-source software packages) and availability of large data sets,^36^ a multitude of data-driven modeling and environmental-economic analyses methods have been utilized to assess the operational, economic, and environmental aspects of implementing an RRCC technology. However, it can be challenging to navigate these various methods and find the most effective ones for RRCC from organic waste streams. Existing review articles contain content such as mechanisms of the different RRCC technologies,^24,37−40^ principles and performance of the different ML methods,^24,41−43^ different stages of the LCA framework,^40,44−46^ and different methods to conduct LCA and their outputs.^38−40,44^ In addition, these review articles either focused on data-driven process modeling or LCA and LCCA/TEA in RRCC from a specific organic waste or a group of similar organic waste streams (e.g., based on wetness or dryness) through the utilization of a single technology (see Table S1 for details). However, considering the large number of studies in the past two decades, a need exists for unified reporting and comparison of such relevant information to appropriately inform practitioners about the effective utilization of data science tools for sustainable RRCC from organic waste streams. Further, although recent studies have demonstrated the potential of integrating multiple technologies to maximally utilize organic waste and recover a variety of resources,^13−15,47,48^ a literature review of data science applications in such integrated RRCC technologies has yet to be conducted.

The overall goal of this study is to conduct a critical literature review covering the wide umbrella of data science applications that includes data-driven process modeling along with the environmental and economic impact assessments in RRCC from multiple organic waste streams through single or multiple-treatment technologies. Specific objectives of the study are to (1) identify the different data science methods used to evaluate RRCC technologies in the literature; (2) investigate the trends, common practices, and challenges for applying these data science methods in RRCC; and (3) formulate recommendations for the effective utilization of the different data science methods for RRCC applications. This work aims to identify critical knowledge gaps and better inform future research in terms of selection, development, and application of the different data science methods for RRCC from organic waste streams. The results from this review can facilitate future research on unexplored aspects of data science applications in RRCC from organic waste and inform practitioners about the effective utilization of various data science methods for implementing RRCC technologies at full scale.

## 2 Methods

### 2.1 Scope of the Literature Review

**Figure 1:**
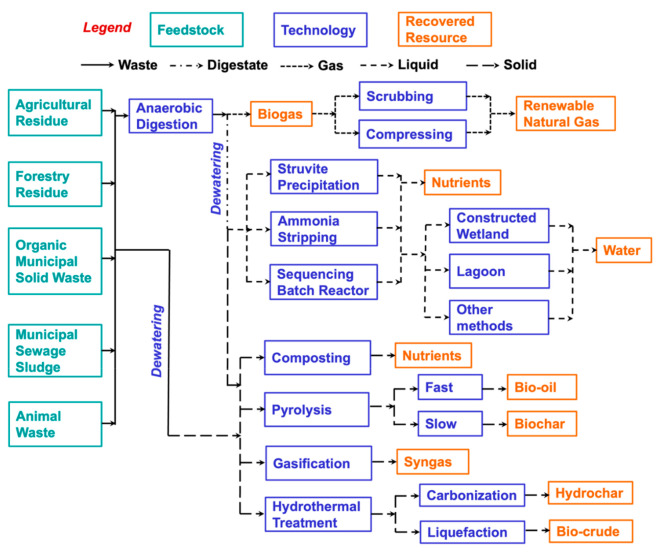
Treatment train comprising organic waste streams, resource recovery and carbon capture (RRCC) technologies, and recovered resources. Other methods for treating liquid effluents from nutrient recovery processes include sequencing batch reactor, reverse osmosis, and tertiary treatment. Dewatering of digestate from anaerobic digestion typically involves mechanical separation, whereas dewatering of feedstocks prior to composting and thermochemical conversion can be done by mechanical separation and conventional heating.

The scope of this literature review included the different types of organic waste streams (hereafter referred to as feedstocks) utilized, the RRCC technologies employed, the recovered resources, and the data science methods employed for process modeling as well as environmental and economic impact assessments. Based on this scope, we used different combinations of keywords in the form of “feedstock” AND “treatment technology” AND “recovered resource” AND “data science tool” in the Google Scholar search bar to find relevant studies (Table S2). For example, to search for studies that applied ML methods to model pyrolysis and recover carbon as biochar from agricultural residue, the keywords “agricultural” AND “pyrolysis” AND “machine learning” AND “biochar” were used. To review articles on integrated technologies, we included all the keywords of the associated technologies. LCA and LCCA/TEA studies in which municipal sewage sludge was first treated via anaerobic digestion and then struvite precipitation was used to treat the liquid digestate for nutrient recovery were found by typing “sewage sludge” AND “anaerobic digestion” AND “struvite” AND “life cycle” AND “nutrients”. Although many of the listed keywords do not include the complete term of a feedstock or treatment technology or data science tool, we found that using concise keywords (e.g., “agricultural” instead of “agricultural residue”) facilitated a more comprehensive literature search. For each combination of keywords, the first 50 peer-reviewed articles (based on year range = “Any time”, sorting = “Sort by Relevance”, and publication type = “Any type”) were checked for desired keywords among the emboldened words in the title and/or brief preview of the article in the search result page. If the desired keywords were present, the abstract was then reviewed to confirm whether the article was within the scope of our review or not. Once confirmed, we reviewed the introduction for citations of previous relevant studies as well as the other main paper sections for pertinent information for the critical review of literature studies on the applications of data science in RRCC. We recorded information from all the articles that were relevant based on our review of keywords in the title, brief preview, and abstract review. We summarized this search in the form a treatment train diagram that illustrated the potentially different pathways by which various resources can be recovered from the feedstocks utilizing these technologies as typically found in the literature (Figure 1). The different feedstocks, technologies, and recovered resources in the treatment train along with the various data science methods utilized are briefly discussed in the following subsections.

#### 2.1.1 Feedstocks

The types of feedstocks for RRCC typically include agricultural and forestry residues, organic municipal solid waste, municipal sewage sludge, and animal waste. Agricultural residues comprise crop residues (e.g., silages of maize and rye, stalks of corn and quinoa, straws of rice and wheat) and food processing residues (e.g., kernel shells and empty fruit bunches from olive and palm oil mills, tomato residues, sugar cane bagasse and straw). Forestry residues are woody waste such as wood chips, stems, barks, leaves, and branches of both hardwood (e.g., eucalyptus) and softwood (e.g., pine) trees as byproducts from forest thinning, timber harvesting, and sawmill operations. Organic municipal solid waste includes food waste from households, restaurants, and hotels in addition to organic waste from markets, green waste (e.g., garden waste, yard trimmings), mix paper, and cardboards. Municipal sewage sludge is composed of the sludge from primary and secondary treatment in wastewater treatment plants. Manure and/or slurries of cattle, pigs, and poultry refer to animal waste.

#### 2.1.2 Technologies and Recovered Resources

The technologies utilize various physical, chemical, and biological processes to recover resources and capture carbon from the feedstocks. For example, anaerobic digestion converts biodegradable organic waste into biogas inside fermentation tanks in an oxygen-free environment.^38,39^ Biogas is typically used to generate heat or electricity or combined heat and electric power where the heat obtained from the combustion of biogas is utilized in a power generation unit to produce electricity.^49,50^ However, in recent studies biogas was upgraded through scrubbing or compression to acquire renewable natural gas for transportation fuel.^51,52^ In addition, anaerobic digestion can also be used to recover biochemicals such as bioethanol as a clean alternative for transportation fuel and lactic acid as a value-added product required by pharmaceutical and chemical industries.^53^

The digestate from anaerobic digestion can be mechanically separated into liquids and solids to recover other resources.^54^ The nutrient-rich liquid digestate can be used to recover nitrogen (N) and phosphorus (P) via struvite precipitation. In this precipitation process, the nutrient-rich digestate, in the presence of magnesium (Mg) and a base, can be converted into struvite (MgNH_4_PO_4_·6H_2_O) fertilizer.^20,55^ Another method that can be applied to recover N from liquid digestate is ammonia stripping. In this process, ammonia is transferred into a gaseous phase at high temperature and pH (i.e., stripping) followed by its absorption to an acid (e.g., sulfuric acid or nitric acid) for recovering ammonium fertilizer (e.g., ammonium sulfate or ammonium nitrate).^20,56^ The liquid digestate can also be treated by a nitrification-denitrification process for biological N removal in sequencing batch reactors.^57,58^ The treated sequencing batch reactor effluent can be used as an organic fertilizer.

The liquid effluent from nutrient recovery technologies can be further treated by low-cost wastewater treatment technologies like constructed wetlands or lagoons to reuse the treated water in crop irrigation or aquaculture.^13,59^ Constructed wetlands and lagoons are considered low cost as they are less expensive to construct, operate, and maintain than conventional mechanical wastewater treatment technologies.^60^ In constructed wetlands, a shallow flow of effluent above the rooted soil surface (or vertical and/or horizontal flow through the planted filter media) removes pollutants via multiple simultaneously occurring processes like sedimentation, filtration, sorption, oxidation–reduction, microbial degradation, and plant accumulation.^61^ The effluent can be treated in lagoons through decomposition of organic matter by bacteria, where the aerobic bacterial action is accelerated by supplying oxygen either by mechanical aeration or introducing algae with the help of naturally occurring wind.^62^

The nutrient-rich solid digestate from anaerobic digestion can be treated by composting, a low-cost process in which organic matter is decomposed by bacteria, fungi, and worms under adequate temperature and time that reduces the weight and volume of the organic waste.^63^ At the same time, composting can inactivate pathogens and produce a valuable soil amendment. Thermochemical conversion of the solid digestate into value-added products is commonly done by pyrolysis, gasification, and hydrothermal treatment. Pyrolysis induces the thermal (i.e., pyrolytic) decomposition of organic matter at 350–800 °C in the absence of oxygen.^64^ Slow-heating produces biochar that can be used for soil amendment and carbon capture, and fast-heating yields bio-oil used as biofuel.^38,39^ Gasification transforms solid and dry biomass into synthesis gases (i.e., syngas that includes carbon dioxide, carbon monoxide, and hydrogen) used as biofuel at temperatures above 700 °C in the presence of gasifying agents like air, oxygen, steam, carbon dioxide, or a combination of the agents.^38,39^ Hydrothermal treatment is conducted using hot pressurized water where hydrochar for soil amendment (among other applications such as solid fuel, pollutant removal from wastewater. etc.) is obtained through carbonization at a temperature of 180–250 °C, pressure of 1.2–2.5 MPa, and residence time of 2–16 h, whereas liquefaction results in biocrude used as biofuel at temperatures of 250–500 °C, pressures of 5–35 MPa, and residence times of 5–60 min.^38,39^ In addition to treating the solid digestate, composting and thermochemical conversion technologies are also used to directly treat feedstocks for RRCC. In some cases, the relatively wet organic waste streams are pretreated (e.g., moisture reduced by dewatering) to improve the efficiency or full functioning of the technologies.

#### 2.1.3 Data Science Tools for Process Modeling

Data-driven methods applied in RRCC comprise statistical and ML models. Typically, statistical methods in RRCC are utilized for inference where data is fitted based on statistical assumptions (e.g., normal distribution, homoscedasticity, etc.) to characterize input-output relationships, sometimes in the form of an explicit mathematical equation.^65^ The coefficients in the equation represent the relationships between the input and output variables. ML methods, on the other hand, are applied for the prediction of the processes of a technology where algorithms drawn from statistical techniques (e.g., bootstrapping, logistic regression, etc.) are utilized iteratively to learn input-output relationships hidden within the data to produce the most accurate model.^65^ Although ML methods do not provide an explicit mathematical equation like statistical methods to explain the relationships between input and output variables, significant progress has been made over the years for developing methodologies (e.g., Shapley values, local surrogate models) to make the ML predictions interpretable.^66,67^ ML methods can be more complex compared to statistical methods as they require large data sets and entail computationally demanding operations such as data preprocessing, hyperparameter tuning, iterative refinement, and cross-validation.^68−70^ Overall, although the goal of applying statistical and ML methods can often be similar, based on the complexity of the underlying processes and requirement of interpretability, the general goal of applying statistical methods in the RRCC literature have been to understand how a technology behaves with respect to variations in input data, whereas applications of ML methods aimed to achieve the best possible prediction of certain attributes of interest, classification of data samples, or clustering/grouping of samples (as related to a given technology), utilizing varying input data.

These statistical and ML models are driven by either primary or secondary data. Primary data can be obtained from laboratory experiments, field experiments, or running numerical experiments in process simulation software like Aspen Plus.^71^ Secondary data are generally a compilation of experimental data sets obtained from previously conducted studies.^71^ Primary or secondary data are commonly analyzed using statistical methods (e.g., multiple linear regression (MLR), partial least-squares regression (PLSR), and multiple polynomial regression (MPR)) (Table 1). More advanced ML methods for regression are starting to gain traction (e.g., artificial neural network (ANN), adaptive neuro fuzzy inference system (ANFIS), support vector regression (SVR), regression tree (RT), random forest regression (RFR), extreme gradient boosting (XGBoost), and Gaussian process regression (GPR)) (Table 1).^24,41,42^ Although variations of these methods are also used for classification, this topic was not within the scope of our review. Some examples of classification in RRCC include the use of random forest and *k*-nearest neighbors to classify low, medium, and high ranges of biogas production in anaerobic digestion based on the combination of operational data and microbial community,^72,73^ and the use of tree-based ML methods to classify gaseous, liquid, and solid phases of pyrolysis output utilizing operational data.^74^ Similarly, although unsupervised machine learning techniques such as *k*-means clustering have been applied to some feedstock supply related problems in RRCC (e.g., anaerobic digestion^75,76^ and pyrolysis^77^), such techniques are not included in this review.

**Table 1:** General Information on Data Science Methodologies Typically Used in the RRCC Literature

statistical and machine learning methods		
method	type and algorithm	general uses in RRCC
Multiple Linear Regression (MLR)^78^	Statistical-Linear	Inference
Partial Least Squares Regression (PLSR)^78^	Statistical-Linear	Inference
Multiple Polynomial Regression (MPR)^79−82^	Statistical-Nonlinear	Inference and optimization
Artificial Neural Network (ANN)^83^	ML-Neural network	Prediction
Adaptive Neuro Fuzzy Inference System (ANFIS)	ML-Neural network	Prediction
Regression Tree (RT)^84^	ML-Tree-based	Prediction
Random Forest Regression (RFR)^84^	ML-Tree-based	Prediction
Extreme Gradient Boosting (XGBoost)^85^	ML-Tree-based	Prediction
Support Vector Regression (SVR)^86,87^	ML-Kernel-based	Prediction
Gaussian Process Regression (GPR)^88^	ML-Kernel-based	Prediction

life cycle assessment methods^b^		
method	developer	general environmental impact categories used in RRCC^a^
Centrum voor Milieukunde Leiden (CML)^89^	Leiden University, Netherlands	GWP, EP, AP, HTP, ETP, ODP, Smog, RDP, LU
Eco-Indicator^90^	PRé Sustainability, Netherlands	GWP, EP, AP, HTP, ETP, ODP, RDP, LU
Environmental Development of Industrial Products (EDIP)^91^	Danish Environmental Protection Agency	GWP, EP, AP, HTP, ETP, ODP, Smog, RDP
Intergovernmental Panel on Climate Change (IPCC)^92^	IPCC	GWP, EP, AP
RIVM, CML and, PRé Consultants (ReCiPe)^93^	RIVM, Radboud University Nijmegen, Leiden University and PRé Sustainability, Netherlands	GWP, EP, AP, HTP, ETP, ODP, Smog, PMFP, RDP, LU
Tool for the Reduction and Assessment of Chemical and Other Environmental Impacts (TRACI)^94^	United States Environmental Protection Agency	GWP, EP, AP, HTP, ETP, ODP, Smog, RDP
International reference Life Cycle Data System (ILCD)^95^	European Union	GWP, EP, AP, HTP, ETP, ODP, Smog, PMFP, RDP, LU
Impact 2002+/World+^96,97^	Quantis (Europe and United States)	GWP, EP, AP, HTP, ETP, ODP, RDP, LU
Greenhouse gases, Regulated Emissions, and Energy use in Technologies (GREET)^98^	Argonne National Laboratory, United States	GWP

a Note: GWP = Global warming potential, EP = Eutrophication potential, AP = Acidification potential, HTP = Human toxicity potential, ETP = Eco-toxicity potential, ODP = Ozone depletion potential, PMFP = Particulate matter formation potential, RDP = Resource depletion potential, LU = Land use.

b This list consists of methods most used in RRCC. The complete list is provided in Table S3.

Data-driven methods follow different algorithms for modeling purposes. MLR and PLSR are both linear regression models, where PLSR models are able to resolve the colinearity among the input variables and provide unbiased input-output relationships.^78^ On the contrary, MPR is nonlinear in nature and typically represents a second- or third-order polynomial regression model in the RRCC literature.^79−82^ The outputs of MPR are further utilized for process optimization through response surface methodology. Among ML models, ANNs are black-box models that mimic biological neurons by attempting to replicate information transfer between biological neurons through electrical signals using mathematical functions.^99,100^ ANN models have been highly successful in combining complex nonlinear input data to predict outputs.^101^ ANFIS combines ANN and human knowledge using fuzzy if-then rules to establish the input-output relationship within a data set to fit data from nonlinear systems.^83^ RT methods utilize decision trees for regression that are based on a heuristic modeling approach where input-output relationships are constructed using multiple models that branch and are informed from each other.^84^ Both RFR and XGBoost are tree-based ML models. The RT models in RFR are built independently (i.e., all trees at the same time, or bagging) where the final model represents the average results from all the RTs.^84^ In XGBoost, the RT models are built sequentially (i.e., one tree at a time), where the results of one model informs the improvement of the following model (i.e., boosting) by minimizing the gradient of the loss function.^85^ Unlike other ML methods, the tree-based methods have the added advantage of providing feature importance that represents the weight of each input variables to predict the output, which is also important for explainability.^24^ SVR is a support vector machine used for regression that utilizes the kernel method where nonlinear functions map the input-output data into a high dimensional space and finds a predictive function from which all data points are within a tolerable distance.^86,87^ GPR utilizes the kernel method to construct input-output relationships by incorporating Gaussian distribution over a regression function space and subsequently updating the function with the data set.^88^ Regardless of their algorithms, a common theme for most data-driven methods is the ability to successfully identify relationships (both linear and nonlinear) within input-output data sets. Therefore, these modeling tools are highly suitable for identifying the complex interactions in the physical, chemical, and biological processes involved in the RRCC technologies.^24,102^

#### 2.1.4 Data Science Tools for Environmental and Economic Impact Analyses

LCA is performed in four stages: goal and scope definition, life cycle inventory (LCI) analysis, impact assessment, and interpretation.^103,104^ The goal and scope comprise defining the functional unit representing the performance of RRCC in terms of the amount of resource recovered or feedstock managed and setting up the system boundary that includes life cycle material and energy flow paths and the associated processes within the RRCC facility. LCI analysis provides the description of the material and energy flows as well as the associated processes utilizing a combination of primary, literature, and inventory data. Here, primary data are typically obtained from laboratory, field experiments, or RRCC facilities; literature data (sometimes referred to as secondary data) are gathered from previous studies with similar objectives; and inventory data, such as Ecoinvent, represent a comprehensive compilation of data pertinent to the processes of the associated technologies.^105^ In the impact assessment stage, characterization factors are applied to the LCI analysis results to quantify the environmental impacts. Characterization factors are weighting factors that unify all relevant substances resulting from the RRCC processes that contribute to specific environmental interventions called impact categories. The impact categories that are typically reported in the RRCC literature are global warming potential, eutrophication potential, acidification potential, ozone depletion potential, photochemical ozone formation potential (also called smog potential), particulate matter formation potential, human toxicity potential, ecotoxicity potential, land use, and resource depletion potential. The characterization factors for the different impact categories are provided in the various LCA methodologies (Table 1, Table S3).^89−98,106−112^ In general, majority of the LCA methods were either developed by academic institutions or environmental consultants in Europe, except for TRACI (Tool for the Reduction and Assessment of Chemical and Other Environmental Impacts) and GREET (Greenhouse gases, Regulated Emissions, and Energy use in Technologies), which were developed in USA (Table 1). In the RRCC literature, ReCiPe (RIVM, CML and, PRé Consultants) and ILCD (International reference Life Cycle Data System) reported the most (10) impact categories, followed by CML (Centrum voor Milieukunde Leiden), which reported 9 categories (Table 1). Eco-Indicator, EDIP (Environmental Development of Industrial Products), TRACI, and Impact 2002+/World+ reported 8 impact categories. Although Intergovernmental Panel on Climate Change (IPCC) is mostly known for computing global warming potential, some RRCC studies also used it to compute eutrophication and acidification potential. In the final stage, the quantified values of the impact categories are interpreted, where the outputs from the impact assessment stage are systematically evaluated to develop appropriate recommendations for the analysis. Additionally tools can be used in the interpretation stage such as uncertainty, sensitivity, and scenario analyses.^113^ Considering a variety of data used in LCA and LCCA/TEA (e.g., primary, literature, and inventory data), uncertainty analysis is applied to estimate uncertainties related to temporal, geographical, and technological variabilities in the data. Therefore, uncertainty analysis facilitates characterizing indicators, identifying drivers, and setting targets. Sensitivity analysis is used to investigate the degree to which a specific input parameter (e.g., operational, contextual) impacts the outcomes or indicators of LCA and LCCA/TEA. Scenario analysis assesses alternatives to inform deployment of an RRCC technology based on specific assumptions about additional contexts.

LCCA/TEA of RRCC involves the economic evaluation of the material and energy balances and the associated processes obtained from LCA using values from a combination of primary, literature, and inventory data for construction, transportation, interest rate, labor costs, etc.^46^ The most common methods to conduct LCCA/TEA are net present value (NPV), internal rate of return (IRR), payback period (PBP), and return on investment (ROI); additionally, minimum selling price (MSP) and levelized cost of energy (LCOE) were also used in some RRCC studies.^46,114−116^ These methods are quantified utilizing capital expenditure (CAPEX), operation and maintenance (O&M) cost (including energy cost), and revenues. In some studies, energy cost is reported separately from the O&M cost. Additionally, construction cost, collection and transportation cost, disposal cost, and avoided impacts cost can also be included based on data availability. These methods provide the foundation for conducting LCCA/TEA to assess the economic performance of a process designed for resource recovery.^114^ NPV is determined by computing the net cash flow for RRCC (typically O&M costs minus revenues) discounted by the future value of the recovered resource at a constant rate of return over a specified time-period.^117^ A positive value of NPV refers to profit toward an investment. The goal of IRR is to understand the potential profitability of an investment over the time-period that is computed by setting NPV = 0 and determining the discounted rate of return.^118^ PBP is calculated by dividing CAPEX by the annual cash flow and represents the time needed to recover the funds expended from CAPEX.^119^ Therefore, a long PBP would not be acceptable for most traditional investors. ROI evaluates the performance of profitability and is calculated by taking the ratio of net profit over the lifetime of the RRCC facility to CAPEX. MSP is the break-even selling price of the recovered resource (i.e., when NPV = 0). LCOE is used to determine the cost of energy production over the operational life of the RRCC facility.^120^

### 2.2 Collection and Analysis of Literature Information

Our literature search resulted in a total of 616 peer-reviewed articles on the applications of data science in RRCC from feedstocks during 2002–2022. It should be noted that after initial submission in January 2023, some newer data-driven approaches have been published^121−123^ but could not be included in our analysis in this review. Of the 616 articles, 199 were on data-driven process modeling until April 2022, 414 were on the use of LCA and LCCA/TEA until June 2022, and three were on the use of data-driven process modeling with LCA and LCCA/TEA (Tables S4 and S5). Among 80 different peer-reviewed journals, 36% of the data-driven modeling studies were published in “Bioresource Technology”, whereas 73% of the LCA and LCCA/TEA studies were published in “Journal of Cleaner Production” across 90 different journals (Tables S6 and S7). In general, we gathered information on the title, author list, publication year, journal name, feedstock, RRCC technology, recovered resource, and study area. In addition to the general information, the data-driven modeling studies were reviewed to collect size and source of data, data preparation strategies, type of data-driven model applied, type of application, identification methods for selecting input features, input features and output variables of the model, and the performance evaluation of the model (Table S4). The LCA and LCCA/TEA articles were reviewed to collect the source of LCI data, software used, LCA and LCCA/TEA methods, environmental and economic impact factors reported, and type of interpretation method (Table S5).

We divided the twenty-year time period of 2002–2022 into five separate bins with an equal number of years in each bin (i.e., 2002–2005, 2006–2009, 2010–2013, 2014–2017, and 2018–2022) to investigate the trends in data science applications in RRCC. For each of these bins, we computed the number of applications (hereafter referred as frequency) of statistical versus ML methods and LCA versus LCCA/TEA. We computed the relative frequencies of the practices followed at the different stages of developing the statistical and ML methods for process modeling to explore knowledge gaps and indicate popular approaches and best practices in the data science pipeline. Besides exploring the use of the different LCA methods over time, we also computed their frequency in different countries. To identify knowledge gaps with respect to the various RRCC technologies, we computed the frequency of the data-driven methods along with LCA and LCCA/TEA and summarized the information using Sankey diagrams. Current practices of data-driven modeling in RRCC were investigated by determining the frequency of the input variables utilized to develop the models of the different technologies. The number of total input variables ranged between 2 and 24 for the different technologies utilized across the literature (Table S8). To summarize this information, we calculated the relative frequency of each input variable by normalizing their respective frequency value with the maximum value for each technology. The relative frequencies were then classified as high (0.75–1), moderate (0.5–0.75), and low (0.5–0) to distinguish the popularity of the input variables. For example, to model anaerobic digestion, the input variable with the maximum frequency was feedstock quantity (29) in the reviewed literature (Table S8). To determine the relative frequency of temperature as an input variable to model anaerobic digestion, its frequency (21) was divided by the frequency of the feedstock quantity (21/29 = 0.72). Therefore, based on the classified ranges, using temperature as a model input for anaerobic digestion had high popularity based on the literature. To indicate the current practices of LCA and LCCA/TEA, we computed the relative frequency of the data sources with respect to different RRCC technologies along with the different environmental and economic impact indicators. Additionally, the frequency of the different interpretation methods in LCA and LCCA/TEA were analyzed over the previously specified time periods to observe the trend of the application of sensitivity, uncertainty, and scenario analyses for the interpretation of the environmental and economic impact indicators.

## 3 Applications of Data Science in RRCC from Organic Waste Streams

**Figure 2:**
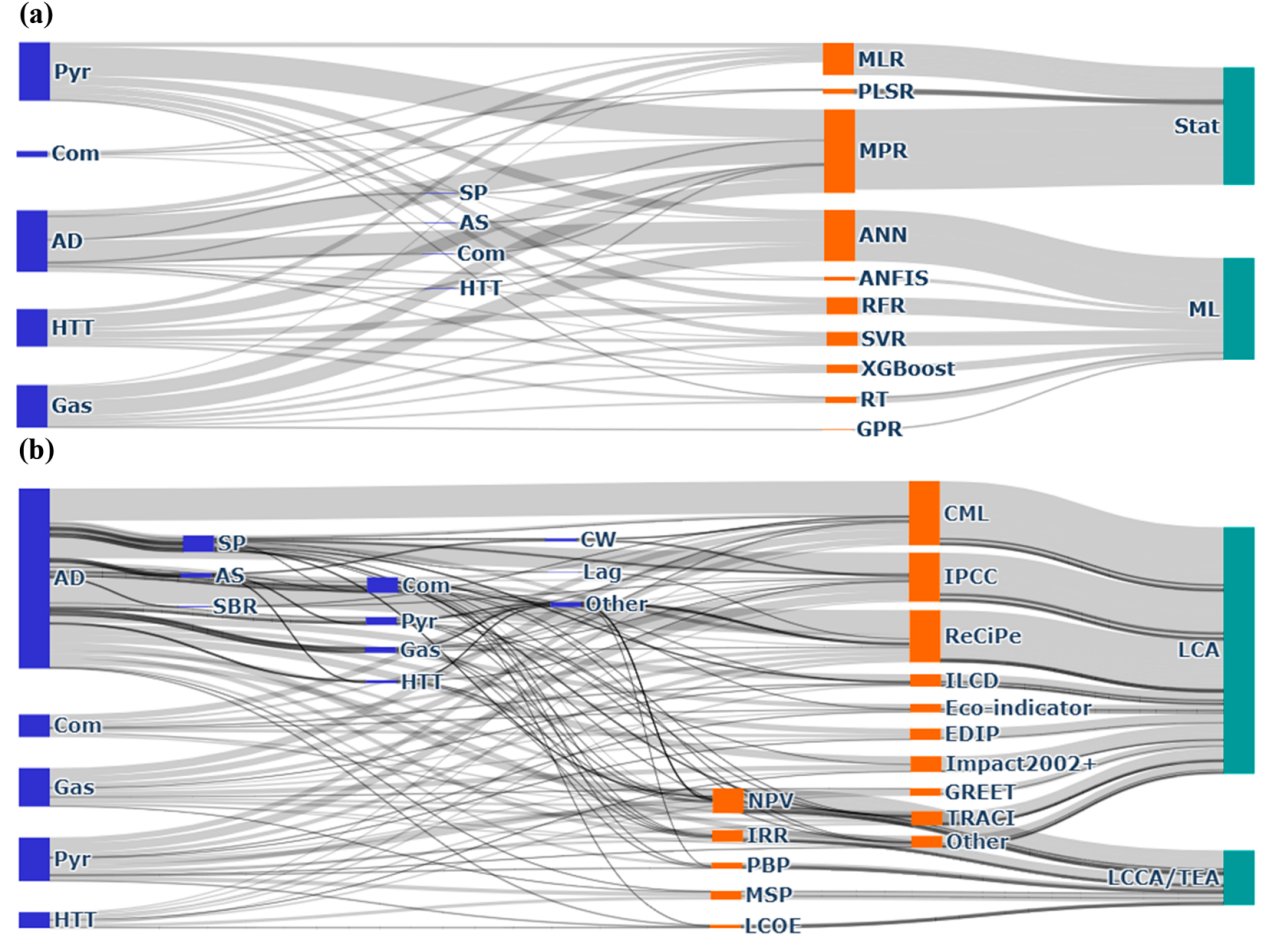
Proportions of (a) data-driven methods for process modeling and (b) life cycle assessment (LCA) and life cycle cost analysis/techno-economic analysis (LCCA/TEA) in technologies or combination of different technologies employed for transforming various feedstocks into resources found in literature during 2002–2022. (Notations: AD = anaerobic digestion, Com = composting, Gas = gasification, Pyr = pyrolysis, HTT = hydrothermal treatment, SP = struvite precipitation, AS = ammonia stripping, SBR = sequencing batch reactor, CW = constructed wetlands, Lag = lagoon, Other = reverse osmosis, SBR, and tertiary treatment.)

The percentage of applications of the different statistical and ML methods for process modeling (Figure 2a) and the various LCA and LCCA/TEA methods for environmental and economic impact analyses (Figure 2b) corresponding to the RRCC treatment train were summarized in Sankey diagrams (see Tables S9 and S10 for the values in Figure 2, panels a and b, respectively). The applications of these data science methods are compared utilizing their common characteristics in the following sections.

### 3.1 Statistical and ML Methods for Process Modeling

#### 3.1.1 Anaerobic Digestion

Of the RRCC technologies reviewed in the literature, 28% of the data-driven modeling applications involved predicting biogas yield or biomethane potential from anaerobic digestion. Of the data-driven anaerobic digestion studies, 49% utilized statistical methods and 51% utilized ML methods (Figure 2a and Table S9). The linear regression models (MLR and PLSR) were 11% of all data-driven methods applied to model anaerobic digestion processes. These methods were applied using feedstock properties (e.g., lignin, cellulose, hemicellulose content) as model input from both primary and secondary data for agricultural residue and animal waste.^124−131^ Apart from the limited applications of linear models, 39% of the data-driven anaerobic digestion models were based on MPR,^28,79,129,132−157^ and 41% were ANN and ANFIS^28,129,131,137,153−175^ (Figure 2a and Table S9). These models used primary data comprised of operational parameters (e.g., temperature, hydraulic retention time) as well as the quantity of feedstocks (agricultural residue and animal waste). Among different ML methods, ANN,^102,176^ SVR,^102,176,177^ RFR,^102,176^ and XGBoost^102,176,177^ were evaluated to predict biogas yield from organic municipal solid waste and sewage sludge. These studies used both primary and secondary data of various feedstock properties (e.g., total and volatile solids, chemical oxygen demand, pH) for model development. Further comparison of these different ML methods using secondary data of other feedstocks (e.g., agricultural residue and animal waste) with operational parameters besides feedstock properties could be used to more accurately evaluate their effectiveness to model anaerobic digestion processes.

#### 3.1.2 Treatment of Liquid and Solid Digestate

Only 4% of the data-driven modeling applications for RRCC reviewed in the literature focused on the treatment of liquid and solid digestate from anaerobic digestion, all of which were statistical methods (Figure 2a and Table S9). The lack of ML applications could be perhaps that such RRCC practice is still emerging, and therefore large data sets do not exist in the literature for developing complex ML models. Among such studies, 2% applied MPR to predict nutrient (N and P) recovery and ammonia removal efficiency of struvite precipitation and ammonia stripping, respectively, utilizing pH and molar ratio (e.g., Ca^2+^/PO_4_^3–^, Mg^2+^/PO_4_^3–^, and NH_4_^+^/PO_4_^3–^) experimental data (Figure 2a and Table S9).^178−183^ These models used liquid digestate derived from animal waste and organic municipal solid waste as feedstocks. PLSR models were developed by one study to model N and P recovery from the solid digestate composting of multiple feedstocks (i.e., agricultural residue, animal waste, organic municipal solid waste, and sewage sludge) using primary data of the C/N ratio and extractable compounds in water.^184^ MPR was used to predict biocrude yield via hydrothermal liquefaction from solid digestate of agricultural residue and animal waste using temperature, time, and solvent/biomass ratio.^185^ MLR was applied by two studies for predicting hydrochar yield through hydrothermal carbonization from solid digestate of agricultural residue utilizing temperature and reaction time.^186,187^ Based on the literature reviewed, MPR still remains untested to model solid digestate treatment processes such as composting, pyrolysis, gasification, and hydrothermal carbonization. Of the studies reviewed, none included statistical or ML methods to model pyrolysis and gasification of solid digestate. Lastly, RRCC literature lacks the application of data-driven methods to model treatment of liquid effluent from the liquid digestate treatment processes utilizing low-cost wastewater treatment technologies. Overall, statistical methods have been limitedly utilized to model RRCC from either solid or liquid digestates, whereas ML has yet to be tested for such applications.

#### 3.1.3 Composting

Applications of data-driven methods to model composting processes were only 3% (75% statistical and 25% ML methods) of the applications reviewed from the literature (Figure 2a and Table S9). These studies used primary data from composting experiments and applied MLR,^188−190^ PLSR,^191^ MPR,^192,193^ and ANN^189,193^ to model N and P content from composted animal waste. These models were developed using operational parameter (composting temperature) and feedstock properties (C/N ratio and electrical conductivity) as model inputs. These limited studies on the modeling of composting processes suggest that other ML methods need to be tested using secondary data to evaluate their effectiveness.

#### 3.1.4 Pyrolysis

Pyrolysis models comprised 28% (57% statistical and 43% ML methods) of the data-driven applications for modeling the different RRCC technologies reviewed in the literature (Figure 2a and Table S9). Among the applications of data-driven methods in pyrolysis modeling, 49% developed MPR models to recover biochar or bio-oil from dry feedstocks using both primary and secondary data of operational parameters (Figure 2a and Table S9).^27,80,194−223^ Operational parameters of the data-driven pyrolysis models typically included temperature, heating rate, gas flow rate, and residence time. Only 8% of the pyrolysis data-driven models were based on MLR (Figure 2a and Table S9). One such study used primary data comprising operational parameters and feedstock quantity to predict biochar yield.^224^ Other studies compared between MLR and MPR to predict bio-oil and biochar yield from the dry feedstocks using secondary data with previously mentioned model inputs.^225,226^ Studies using secondary data (with operational parameters and feedstock properties) further demonstrated that MLR models of pyrolysis were generally outperformed by different ML models, namely ANN,^226^ RT,^227^ XGBoost,^226^ RFR,^227,228^ and SVR.^227^ Among the different ML models of pyrolysis, ANN and ANFIS were developed with operational parameters and feedstock properties using both primary data and secondary data.^229−234^ In comparative studies, ANNs developed with just operational parameters of pyrolysis seemingly provided better predictions of bio-oil and biochar yield than MPR when using secondary data.^235,236^ SVRs developed with secondary data representing operational parameters and feedstock properties provided relatively improved performance compared to the ANNs for both dry and wet feedstocks (animal waste, agricultural and forestry residue).^237−239^

In separate studies, pyrolysis models using secondary data with similar input variables were developed applying RFR and SVR for agricultural and forestry residue.^240−243^ In a comparative study, RFR performed better than SVR for wet feedstocks (animal waste, organic municipal solid waste and sewage sludge) where secondary data was used to develop both models.^244^ Another study contrasted performances among different ML methods developed using secondary data of operational parameters and properties of the dry feedstocks and found that RFR provided better predictions than SVR, ANN, and XGBoost.^245^ Overall, the findings from this review illustrate that ML methods possess good potential to model pyrolysis processes for bio-oil or biochar from both dry and wet feedstocks utilizing operational parameters and properties of feedstocks.

#### 3.1.5 Gasification

Of the reviewed studies that applied data-driven methods to model RRCC technologies, 20% (36% statistical and 64% ML methods) represented models that predicted syngas yield through the gasification of various feedstocks (Figure 2a and Table S9). Gasification was most frequently modeled by applying MPR^82,246−261^ using primary data and ANN^247,262−276^ using both primary and secondary data. These MPR and ANN models respectively, comprised 33% and 36% of the data-driven gasification models (Figure 2a and Table S9). In addition, these models were developed with operational parameters (e.g., temperature, steam/biomass ratio, equivalence ratio) and properties of dry feedstocks (e.g., moisture, ash, and C–H–O content). In one study where primary data (with operational parameters) was used as model inputs, performance of MLR to model gasification was comparable to ML models (SVR and RT).^277^ However, in another study that used secondary data comprising similar inputs, MLR was outperformed by ANN.^278^ Secondary data that included operational parameters and feedstock properties were also used to model the gasification processes of multiple wet feedstocks (animal waste, organic municipal solid waste, and sewage sludge) using XGBoost.^279,280^ Gasification has been modeled and compared among a variety of ML methods such as ANN,^29,281^ SVR,^29,281,282^ RT,^29,281^ RFR,^29,282^ and XGBoost.^29^ These models were developed using primary data that included operational parameters and properties of agricultural and forestry residue. Among comparative studies that utilized secondary data, syngas yield was predicted employing ANN, GPR, SVR, and RFR.^283^ These model comparisons indicate that ML methods were effective tools to predict syngas yield from gasification of both dry and wet feedstocks.

#### 3.1.6 Hydrothermal Treatment

The data-driven methods applied to model hydrothermal treatment processes and predict biocrude and/or hydrochar yield included 18% (50% statistical and 50% ML methods) of all the applications reviewed in the literature (Figure 2a and Table S9). Among the data-driven models of hydrothermal treatment, 15% were MLR models^284−286^ and 35% were MPR models^81,287−302^ (Figure 2a and Table S9). These models were developed using primary data that involved operational parameters (e.g., temperature, residence time, biomass/water ratio) for multiple feedstocks (organic municipal solid waste, agricultural and forestry residue). When compared with ML methods developed with secondary data, predictive performance of MLR models was lower than that of ANN,^303^ RT,^304,305^ and RFR.^304^ Among the applications of ML methods, hydrothermal treatment processes were modeled by applying ANN^306^ using secondary data, and RFR^307−309^ using both primary and secondary data. These models used properties of multiple feedstocks as model inputs. Multiple studies evaluated different ML methods such as ANN,^29,310^ SVR,^29,276,306,310,311^ RT,^312^ RFR,^276,306,310−312^ and XGBoost^29,306,311,312^ to predict hydrochar and/or biocrude yield. These models utilized secondary data comprising operational parameters and feedstock properties as input variables for model development. Overall, ML methods demonstrated promising results in recent literature to model hydrothermal treatment.

### 3.2 LCA and LCCA/TEA for Environmental and Economic Impact Analyses

#### 3.2.1 Anaerobic Digestion

Applications of LCA and LCCA/TEA to assess the environmental and economic impacts of acquiring biogas from anaerobic digestion comprised 43% of the applications reviewed in the literature (Figure 2b and Table S10). Among these applications, 86% were LCA of which 69% utilized the following methods: CML, IPCC, ReCiPe, ILCD, and EDIP (Figure 2b and Table S10). The majority of these studies (with agricultural residue, organic municipal solid waste, and animal waste as feedstocks) were conducted in European countries (i.e., Italy, Germany, United Kingdom) and Asia (i.e., China, India, Iran).^30,201,313−417^ With these LCA methods, studies compared the environmental impacts of anaerobic digestion with other RRCC technologies. Of such studies, gasification^418−423^ and pyrolysis^421,424^ were compared with anaerobic digestion in the European region (i.e., Sweden, United Kingdom). Further, in China, anaerobic digestion was compared with composting,^425−430^ hydrothermal carbonization,^431^ and gasification^432^ using organic municipal solid waste as the feedstock. The environmental impacts of integrating anaerobic digestion with technologies (i.e., scrubbing, compression) that can upgrade biogas to renewable natural gas were assessed in Europe (i.e., Sweden, Denmark) employing IPCC and ReCiPe.^433−446^ Only 3% of the studies conducting LCA of anaerobic digestion utilized TRACI (Figure 2b and Table S10). Both TRACI and IPCC were preferred over ReCiPe in Canada and the USA to determine the environmental impacts of anaerobic digestion with organic municipal solid waste and animal waste as feedstocks.^447−452^ These preferred methods were also used to compare anaerobic digestion with gasification^453,454^ and composting^454^ in North America. Studies in Brazil, South Africa, and Australia conducted environmental impact analyses of anaerobic digestion and compared between anaerobic digestion and composting by applying CML and ReCiPe using feedstocks of organic municipal solid waste and animal waste.^430,455−473^

Among the LCA and LCCA/TEA studies of anaerobic digestion, 14% were LCCA/TEA, of which 9% utilized NPV and IRR as economic impact analysis methods (Figure 2b and Table S10). Studies in the European and Asian regions conducted LCA of anaerobic digestion with organic municipal solid waste and animal waste as feedstocks primarily applying CML followed by LCCA/TEA that employed NPV and IRR.^474−500^ These LCCA/TEA methods, along with CML, were also used by studies in European and Asian countries to compare anaerobic digestion with composting,^501−503^ gasification,^504,505^ and pyrolysis.^503^ Studies in USA that quantified both environmental and economic impacts of anaerobic digestion applied TRACI and IPCC to conduct LCA and NPV for LCCA/TEA.^51,115,506−513^ Of the studies reviewed, more conducted environmental and economic impact assessments of anaerobic digestion in European countries and China compared to the other regions of the world, particularly South America and Africa. Importantly, CML and ReCiPe were preferred LCA methods globally, although TRACI was preferred in USA. Further, NPV and IRR were the most common economic impact analysis methods for LCCA/TEA.

#### 3.2.2 Treatment of Liquid and Solid Digestate

Studies that assessed the environmental and economic impacts of treating liquid and solid digestate from anaerobic digestion comprised 13% of all the LCA and LCCA/TEA studies reviewed (Figure 2b and Table S10). LCA studies focused on biogas production from anaerobic digestion integrated with treatment of the liquid digestates to recover nutrients via struvite precipitation^514−520^ and ammonia stripping^521,522^ utilizing municipal sewage sludge and animal waste as feedstocks. These studies were conducted by applying CML and ReCiPe in European countries and using TRACI in the USA. By applying ILCD, a study in China compared the environmental impacts of recovering nutrients through struvite precipitation and ammonia stripping from liquid digestate and composting from solid digestate of animal waste.^523^ Studies in Spain, Finland, Hong Kong, and Singapore applied CML, ReCiPe, IPCC, and ILCD to conduct an LCA of treating the solid digestate of organic municipal solid waste via composting^524,525^ and compare composting with gasification^526^ and pyrolysis.^527^ LCA studies that assessed the environmental impacts of pyrolysis^32,528−535^ and gasification^536−538^ of solid digestate from municipal sewage sludge were investigated in China, Italy, and Morocco that applied CML, ReCiPe, and IPCC. These methods were used to assess the environmental impacts of recovering nutrients from solid digestates of wet feedstocks through composting^539−544^ and from their liquid digestates via struvite precipitation^31,545^ and sequencing batch reactors^57,58^ in USA, Germany, Italy, Spain, and Iran. Studies in Germany and China compared the environmental impacts between composting,^546−549^ hydrothermal carbonization,^550,551^ and gasification^551^ of organic municipal solid waste and its solid digestate using different LCA methods. Only 4% of the reviewed studies included LCCA/TEA of treating the digestate from anaerobic digestion (Figure 2b and Table S10). Applications of LCCA/TEA to conduct economic impact analysis of gasifying,^116,552−555^ pyrolyzing^556^ and hydrothermally liquefying^557^ solid digestate of animal waste, and municipal sewage sludge utilized NPV and LCOE.

LCA and LCCA/TEA studies conducted for systems that integrated anaerobic digestion and digestate treatment technologies for resource recovery with wastewater treatment technologies for water reuse were only 2% of the applications reviewed in the literature (Figure 2b and Table S10). In addition to quantifying the environmental impacts of anaerobic digestion of wet feedstocks integrated with treatment of liquid (via struvite precipitation or ammonia stripping) and solid digestate (via composting or gasification), studies in Spain, Italy, Belgium, and United Kingdom used CML, ReCiPe, and IPCC to conduct LCA for treating the liquid effluent via constructed wetlands,^14,59,558^ reverse osmosis,^48,462^ or sequencing batch reactor^47^ for water reuse. Studies in Costa Rica and China assessed anaerobic digestion integrated with struvite precipitation and composting, respectively, for nutrient recovery and lagoon for liquid effluent treatment where animal waste was the feedstock.^13,559^ A study in the USA utilized municipal sewage sludge to assess the integration of anaerobic digestion, pyrolysis, hydrothermal liquefaction, ammonia stripping, and tertiary treatment of effluent using ReCiPe for LCA and NPV for LCCA/TEA.^15^ Generally, fewer LCA and LCCA/TEA studies on the treatment of solid and liquid digestates for RRCC have been completed compared to assessing the environmental and economic impacts of just producing biogas. Of such literature reviewed, CML and ReCiPe were the most applied LCA methods, and NPV was the common LCCA/TEA method. Fewer LCA and LCCA/TEA studies with anaerobic digestion and digestate treatment integrated with effluent treatment for water reuse exist, although geographically these studies are surprisingly spread out.

#### 3.2.3 Composting

Only 8% of the LCA and LCCA/TEA studies reviewed in the literature included the environmental and economic impact analyses of composting (Figure 2b and Table S10). Among these studies (with organic municipal solid waste and animal waste as feedstocks), 59% applied CML, ReCiPe, and EDIP where the majority of studies were conducted in Europe and China (Figure 2b and Table S10).^112,560−574^ These methods and feedstocks were also used in the studies in Australia, Argentina, and Brazil except for the USA where TRACI was the preferred method.^463,575−579^ Only one of these studies used ROI for economic impact analysis. Although these findings suggest that composting has not been substantially assessed for environmental and economic impacts, several LCA and LCCA/TEA studies were conducted to compare between anaerobic digestion and composting as discussed previously (Section 3.2.1).

#### 3.2.4 Pyrolysis

Environmental and economic impact analyses studies of pyrolysis were 15% of the LCA and LCCA/TEA studies reviewed in the literature (Figure 2b and Table S10). 20% of the LCA studies for pyrolysis have been conducted utilizing IPCC (Figure 2b and Table S10). In addition to IPCC, of the pyrolysis studies reviewed, 23% applied CML and ReCiPe in European (e.g., Spain, France, Belgium, Norway, Sweden) and Asian countries (e.g., China, Iran, India) (Figure 2b and Table S10).^580−593^ The environmental impact analysis studies that applied GREET and TRACI were 17% of the pyrolysis studies reviewed and were applied in the USA (Figure 2b and Table S10).^594−623^ These studies (with agricultural and forestry residue as feedstocks) further utilized NPV, IRR, and MSP to conduct LCCA/TEA for economic impact analysis, which were 25% of the LCA and LCCA/TEA studies for pyrolysis (Figure 2b and Table S10). Among other applications of ReCiPe and IPCC, studies in Australia, Chile, and Zambia assessed the environmental impacts of pyrolysis to produce biochar from forestry residue.^223,624−627^ CML and ReCiPe have been used in China, whereas in North America, GREET was applied for LCA and NPV for LCCA/TEA to compare the environmental impacts of pyrolysis using agricultural residue as a feedstock with that of composting, gasification, and hydrothermal treatment.^297,628−635^ Of the studies reviewed, pyrolysis in the USA was assessed for both environmental impacts using GREET and TRACI, and economic impacts using NPV, IRR, and MSP, whereas in other regions of the world, studies mainly focused on environmental impact assessment applying IPCC, CML, and ReCiPe.

We found two studies that integrated statistical or ML methods for process modeling with LCA and/or LCCA/TEA for environmental and economic impact analyses in the literature focused on pyrolysis of organic waste streams. Among such studies, one utilized experimental data from pyrolysis of animal waste in Malaysia to predict both biochar and bio-oil yield utilizing operational parameters and feedstock quantity by applying MLR.^35^ The outputs of the MLR model were then utilized to quantify global warming potential using IPCC, and acidification, eutrophication, smog, and human toxicity potential using CML for the environmental impacts assessment. Another pyrolysis study integrated an RFR model with LCA and LCCA/TEA where the feedstocks were agricultural and forestry residue and municipal sewage sludge.^34^ The RFR predicted biochar yield and energy using secondary data comprised of operational parameters and feedstock properties. Utilizing the predicted outputs, LCA was conducted using GREET to determine the global warming potential, and LCCA/TEA was conducted using ROI and MSP. An important outcome of this study was that the RFR model trained using laboratory-scale data was successfully validated using pilot-scale data. The result demonstrated the ability of ML models to scale-up laboratory-scale operations for RRCC through technical assessment in addition to environmental and economic assessment integrating LCA and LCCA/TEA methods.

#### 3.2.5 Gasification

Like pyrolysis, fewer LCA and LCCA/TEA studies on gasification (13%) exist in the RRCC literature than anaerobic digestion (Figure 2b and Table S10). 39% of these studies applied CML and IPCC for environmental impact analysis focused in Europe, China, and Singapore to produce syngas from agricultural and forestry residue, and organic municipal solid waste (Figure 2b and Table S10).^367,636−661^ Only 6% of such studies conducted LCCA/TEA applying NPV (Figure 2b and Table S10). Applications of TRACI and ReCiPe comprised 19% of the environmental and/or economic impact analyses studies reviewed in the literature. Among these applications, LCA studies applied TRACI for gasifying forestry residue and organic municipal solid waste in the USA (Figure 2b and Table S10).^662−671^ ReCiPe was employed for LCA studies (with agricultural and forestry residue as feedstocks) in South Africa and Latin American regions (e.g., Brazil, Ecuador, Columbia) in addition to using LCOE for LCCA/TEA.^672−678^ Generally, LCCA/TEA of gasification was conducted in fewer studies compared to LCA in the reviewed literature, where the common methods for LCA were TRACI in USA, CML and IPCC in European and Asian Countries, and ReCiPe in the remaining countries.

#### 3.2.6 Hydrothermal Treatment

Even fewer LCA and LCCA/TEA studies (6%) have focused on assessing the environmental and/or economic impacts of hydrothermal treatment compared to pyrolysis and gasification (Figure 2b and Table S10). 35% of these studies were conducted using ReCiPe, IPCC, and ILCD for LCA in Europe (i.e., Germany, Finland, Italy, Sweden), and 10% utilized NPV for LCCA/TEA to acquire hydrochar via hydrothermal carbonization and biocrude through hydrothermal liquefaction (Figure 2b and Table S10).^679−686^ 11% of the hydrothermal treatment studies applied GREET for LCA, and 18% used MSP for LCCA/TEA in the USA to obtain hydrochar and/or biocrude using forestry residue as the feedstock (Figure 2b and Table S10).^687−693^ Among the 8% applications of CML, LCA studies were conducted in Malaysia, China, Brazil, and Chile to assess the environmental impacts of hydrothermal treatment where the agricultural residue was used as the feedstock (Figure 2b and Table S10).^694−697^ Similar to the ML integrated LCA and LCCA/TEA approach observed in two reviewed pyrolysis studies (Section 3.2.4), predictions of hydrothermal treatment were compared among MLR, RT, and RFR where the best predicted outputs were used to conduct an LCA and LCCA/TEA for agricultural and forestry residue in the USA.^33^ These data-driven models were developed using secondary data and included operational parameters and feedstock properties. In addition, the global warming potential was determined using GREET to conduct an LCA with ROI for LCCA/TEA. Of the literature reviewed, GREET was the only LCA method used for assessing the environmental impacts of hydrothermal treatment in USA, although TRACI was the most commonly used method to assess other RRCC technologies. However, like other RRCC technologies, ReCiPe, CML, and IPCC were the most frequently applied LCA methods in European and Asian countries alongside NPV as the LCCA/TEA methods.

## 4 Assessment of the Different Data Science Methods Applied for RRCC

### 4.1 Temporal Progression of the Different Data Science Methods

**Figure 3:**
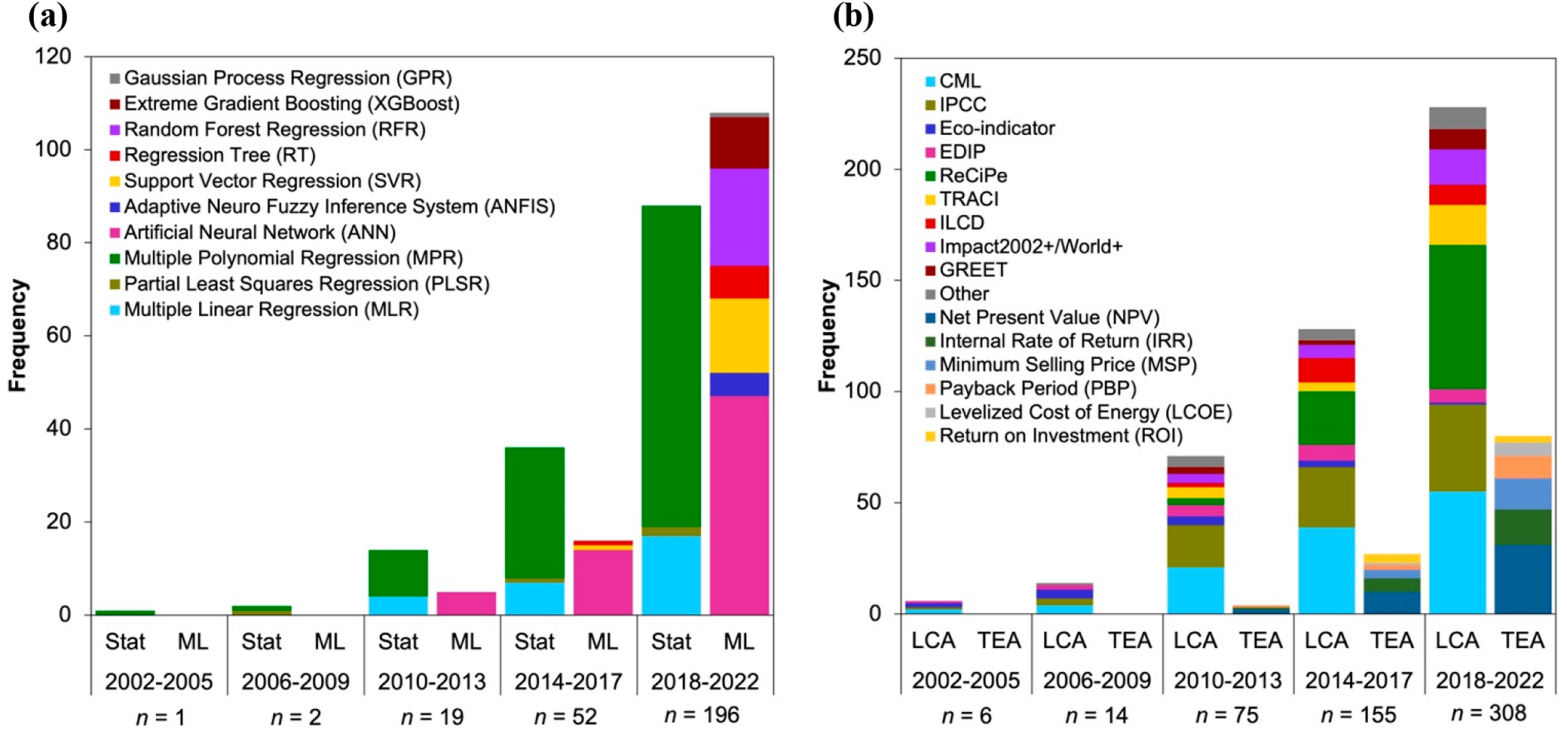
Progression in popularity of data science methods used in RRCC from organic waste streams over the years. (a) Frequency of statistical versus machine learning (ML) methods for process modeling and (b) frequency of life cycle assessment (LCA) versus life cycle cost analysis/techno-economic analysis (LCCA/TEA) for environmental and economic impact analyses over the five time-periods during 2002–2022. The total number of applications in each time-period represented by *n*.

The overall applications of data-driven methods to model the processes in RRCC technologies have increased exponentially during 2002–2022 (Figure 3a). Although researchers began utilizing ML almost a decade later (since 2010), the application of this tool for process modeling has grown at a greater rate than statistical methods over the past several years. Further, analysis of literature data has shown that linear regression models (e.g., MLR and PLSR) have been applied less than nonlinear regression (e.g., MPR) when statistical methods were solely used for process modeling (Figure 3a). This finding is understandable considering the complex nonlinear nature of the chemical and biological processes involved in the treatment of the feedstocks.^698,699^ The application of MPR in RRCC has declined since 2002, possibly due to the increased utilization of ML methods for their enhanced ability to capture complex nonlinear interactions in the organic waste treatment.^245,700,701^ Among the different ML methods, ANNs are the most commonly utilized method since 2010. During 2018–2022, most of the applications of data-driven methods encompassed SVR and the tree-based algorithms such as RT. However, more recently, among the ML models reviewed, RFR and XGBoost have witnessed the fastest growth as the ML model of choice in RRCC technologies (Figure 3a). A review of data-driven models in anaerobic digestion indicated that the developed ANN models exhibited signs of overfitting, likely due to the higher number of hyperparameters compared to tree-based ML methods and the relatively small data sets used for model development.^42^ The review further indicated that among the tree-based ML methods, RFR and XGBoost were more effective than RT to model anaerobic digestion processes, perhaps because these methods are upgraded versions of RT. Therefore, the increased applications of RFR and XGBoost alongside already existing ANN models in the reviewed literature indicated that researchers have begun to explore a multitude of ML methods for effective modeling of RRCC processes.

The application of LCA and LCCA/TEA for RRCC technologies has increased steadily over the years (Figure 3b). Since 2003, a total of 300 peer-reviewed articles were found that apply a variety of LCA methods to assess the environmental impacts of different RRCC technologies. Both LCA and LCCA/TEA were applied by 96 articles, and 23 studies conducted just LCCA/TEA since 2008. Among the different LCA methods, CML and IPCC have been routinely applied over the years (Figure 3b). Both Eco-indicator and EDIP were applied more frequently during 2002–2009. However, applications of these methods reduced quite drastically over the past decade where ReCiPe (an updated version of Eco-indicator)^702^ was predominantly applied. Since 2010, there were notable applications of newer methods like TRACI, ILCD, and GREET.

### 4.2 Important Considerations for Applying Data Science Methods in Process Modeling

**Figure 4:**
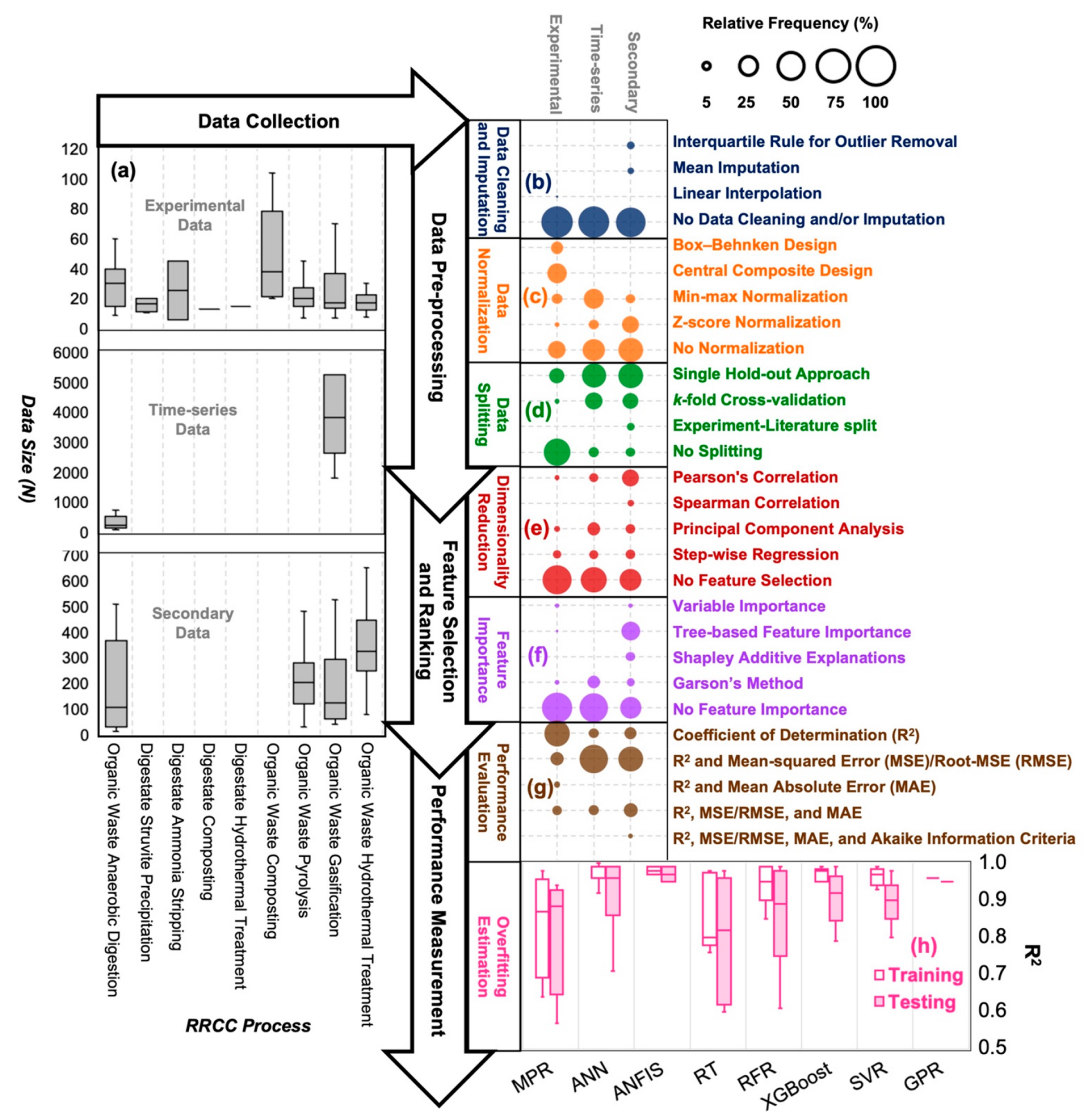
Summary of key considerations for applying data science methods for the process modeling of RRCC technologies at the different stages of model development: (a) type (gray texts) and sample size (gray box and whisker plots) of available data for collection with respect to different treatment processes, data pre-processing (relative frequencies of (b) data cleaning and imputation in blue circles, (c) data normalization in orange circles, and (d) data splitting in green circles), feature selection and ranking (relative frequencies of (e) dimensionality reduction in red circles and (f) feature importance in purple circles), and performance measurement (relative frequencies of (g) performance evaluation measures in brown circles and (h) overfitting estimation example in pink box and whisker plots). The relative frequency refers to the data science applications across all reviewed studies within the categories of experimental, time-series, or secondary data sets.

Based on our review of prior literature, the following subsections represent important aspects to consider while applying data science methods for the process modeling of RRCC technologies at different stages of model development (Figure 4).

#### 4.2.1 Data Collection

##### 4.2.1.1 Primary Data sets: Experimental and Time-series

Among the studies that applied statistical or ML methods for modeling the processes of RRCC technologies, 78% in the reviewed literature utilized primary data sets. More than 90% of these primary data sets were obtained from lab- or pilot-scale experimental setups of various RRCC technologies where the sample sizes of data were relatively small (median *N* = 13–38, where *N* denotes number of data samples) (Figure 4a; gray box and whisker plots). These experimental data sets were commonly prepared utilizing the Box-Behnken Design (*N* = 2*K*(*K* – 1) + *C*) or Central Composite Design (*N* = 2^*K*^ + 2*K* + *C*) where the sample size is dependent on the number of input variables (typically *K* = 2 or 3 in the reviewed studies) and the number of control conditions (typically *C* = 3 or 5 for fractional or full factorial design).^259,703^ A majority of these primary experimental data sets (62%) were used for process optimization through response surface methodology, and the remaining were utilized for prediction (24%) and inference (14%). Contrary to the frequently utilized but small primary experimental data sets, primary time-series data sets representing relatively larger sample sizes from long-term runs in pilot- or full-scale facilities were rarely used (less than 10%) for developing predictive (70%) and optimization (30%) models of only two technologies: anaerobic digestion (median *N* = 233) and gasification (median *N* = 3,831) (Figure 4a; gray box and whisker plots). Time-series data can provide important insights into the real-time nonlinear effects of organic waste input and varying operational conditions of a technology on RRCC output.^704^ Considering that certain RRCC technologies have achieved commercial implementation at large scales (e.g., more than 1,700 anaerobic digestors in the USA alone and close to 100 industrial pyrolysis plants worldwide),^705,706^ limited use of time-series data for data-driven modeling in the reviewed literature reflects the difficulty of researchers to access long-term data from existing RRCC facilities for modeling and analysis. Such inaccessibility to time-series data could also have contributed to the limited applications of deep learning methods in RRCC; only one study was found through this literature review that applied long short-term memory for predicting biogas production in an anaerobic digestion facility.^159^

##### 4.2.1.2 Secondary Data sets

Considering the recent calls for harnessing the underutilized large complex data sets in modern wastewater treatment and resource recovery facilities (via supervisory control and data acquisition systems) to achieve process efficiency and sustainable performance,^707−709^ researchers have been following alternative paths to compile accessible data sets representing larger sample sizes. Since 2015, RRCC studies focused on data-driven modeling have been blending experimental data sets from the literature to develop secondary data sets with relatively greater sample sizes (median *N* = 108–325) for different RRCC technologies although not for integrated RRCC (Figure 4a; gray box and whisker plots). These secondary data sets comprised only 22% of the reviewed studies that applied ML methods for modeling the processes of RRCC technologies, where 82% utilized the data sets for predicting RRCC technology processes and the rest were used for optimization. Considering the current lack of accessible time-series data sets in RRCC, for the time being secondary data sets available in the reviewed literature studies can be used for developing data-driven models of anaerobic digestion (*N* = 509),^177^ pyrolysis (*N* = 419–683),^34,226,233,236^ gasification (*N* = 527),^271^ and hydrothermal treatment (*N* = 325–800)^33,304,311,312^ of various organic waste streams.

#### 4.2.2 Data Pre-processing

##### 4.2.2.1 Data Cleaning and Imputation

In general, data sets collected for modeling and analysis are pre-processed to attain enhanced data quality through cleaning of noisy data and imputation (i.e., filling in) of missing data.^710,711^ However, the data sets utilized in the reviewed RRCC literature rarely (less than 10% of the cases) conducted such pre-processing of the data (Figure 4b; blue circles). Data cleaning and imputation might not have been entirely necessary for the primary experimental data sets (representing 71% of the reviewed literature) because the data preparation under controlled conditions resulted in small but noise-free and relatively complete (i.e., with little or no missing data) data sets. Data cleaning and imputation, however, should have been conducted in the 7% studies that utilized facility-scale primary time-series data sets with relatively larger sample sizes considering that such operations are less controlled and are prone to system damage (e.g., leaks) or sensor failures (e.g., electrical interference) (Figure 4b; blue circles).^707^ Among the 22% studies that utilized secondary data sets, only 7% applied the interquartile range (IQR) rule for outlier removal to deal with noisy data, probably because these are a compilation of experimental data sets prepared under different controlled conditions (Figure 4b; blue circles). The IQR rule is a statistical method which states that any data that lie outside 1.5 times IQR greater than the 75th percentile and 1.5 times IQR less than the 25th percentile of the data set are designated as an outlier. Other statistical methods for detecting outliers (not applied in the reviewed RRCC literature) involve utilizing Z-score normalization (scales data with mean of 0 and standard deviation of 1, and designates any data outside a high cutoff Z-score value as an outlier) and probability density function (estimates probability density function of the data and designates any data having a low estimated probability density as an outlier). The IQR rule has often been applied to treat noise in long-term data in related fields such as wastewater treatment,^712^ streamwater quality,^713^ and household energy consumption.^714^ However, it might be debatable to apply such data cleaning rules considering that the statistically removed outliers might represent extreme but true conditions of the RRCC process and could thereby affect the applicability of the developed model under diverse conditions.^715^ Additionally, ML methods can potentially successfully learn such data due to their pure data-driven nature that is independent from statistical assumptions.^715,716^ Therefore, from a practitioner’s perspective, one effective approach of cleaning data (particularly primary time-series data) could be the use of metadata from sensors, which typically comprise a normal range of values (i.e., minimum and maximum values of a parameter such as temperature that can be measured using the sensor) and measurement accuracy (i.e., maximum difference between actual value of a parameter and the measured value by the sensor), although the accessibility of researchers to a RRCC facility must be taken into account for such case.^717^ Further, missing values in 4% of the secondary data sets were filled utilizing the mean imputation method, whereas one study using a primary experimental data set utilized linear interpolation for this purpose (Figure 4b; blue circles). Other data imputation methods not observed in the reviewed literature are forward or backward imputation and moving average methods, although it should be noted that, for operational data in complex systems such as RRCC technologies, these methods are only effective when the missing data ratio is 1–5%.^711^ Overall, system accessibility and process domain knowledge are essential for cleaning noisy data. Statistical methods, although proven to be useful, should be applied carefully for data cleaning and imputation purposes.

##### 4.2.2.2 Data Normalization

Data normalization was a more prevalent data pre-processing step followed in the reviewed literature than data cleaning and imputation. Data normalization scales or transforms all the input features within a data set from variable magnitudes (e.g., temperature = 250–800 °C and heating rate = 2–25 °C/min in manure pyrolysis) to comparable ranges (e.g., −1 to 1) so that the different features have an equal numerical contribution toward model development.^718,719^ Normalization methods, however, varied in the reviewed RRCC literature based on whether the models were developed using primary or secondary data sets. Based on the defined control conditions of the respective studies, input features of 16% primary experimental data sets were coded as −1, 0, and +1 when using the Box-Behnken Design, and 39% were coded as −α, −1, 0, +1, and +α when using the Central Composite Design (α = (2^*K*^)^1/4^, *K* = number of input variables) for data preparation (Figure 4c; orange circles). Among other studies that utilized primary experimental data sets, 12% applied the min-max normalization (scaling data into −1 or 0 to 1) and 3% applied Z-score normalization (scaling data with mean of 0 and standard deviation of 1) (Figure 4c; orange circles). The min-max normalization method was popular (40%) among studies that used primary time-series data sets for developing data-driven models compared to Z-score normalization (10%) (Figure 4c; orange circles). On the contrary, Z-score normalization was applied more (29%) than min-max normalization (9%) in studies that used secondary data sets for model development (Figure 4c; orange circles). Most importantly, 31% of the reviewed studies with primary experimental data sets, 50% with primary time-series data sets, and 62% with secondary data sets did not normalize their data and therefore might have resulted in biased models (i.e., greater contribution of higher magnitude features to model development than lower magnitude features) (Figure 4c; orange circles).^719^ Past studies on developing accurate ANN and kernel-based ML models like SVR and GPR have exerted strong importance on data normalization.^720,721^ However, for tree-based ML models such as RT, RFR, and XGBoost, whether data normalization should be applicable was debated in one of the reviewed RRCC studies because the tree-based algorithms did not impose assumptions about the data distribution unlike ANN via transfer functions or like SVR and GPR via the kernel functions.^228^ Additionally, tree-based ML models can be insensitive to input data normalization because the order of the numerical features (and not magnitude) are most important for model development.^722^ This could have contributed to the decision of not applying data normalization for developing tree-based ML models, which represented half of the 62% studies using secondary data sets, although the other half representing ANN, SVR, and GPR should also have normalized their data. Overall, a concerning lack of data normalization was observed in the reviewed RRCC literature related to data-driven modeling, and future researchers should integrate this essential data pre-processing step using proven methods like min-max normalization or Z-score normalization (or test both) to develop unbiased models.

##### 4.2.2.3 Data Splitting

The final data pre-processing step followed by the reviewed literature was data splitting for estimating the overfitting of developed models. Data-driven models developed for prediction are prone to overfitting.^723,724^ Overfitting occurs when a model not only fits the true relationship of the input-output data but also the unique noises within the sample data, resulting in a model that performs poorly when applied to predict a different sample of the data.^725,726^ Data-driven models developed in the RRCC literature typically compare the predictive performances during training and testing to identify overfitting. Two strategies have been commonly utilized for training and testing of data-driven models in RRCC: the single hold-out approach and *k*-fold cross validation. In the single hold-out approach (used by 23% experimental, 60% time-series, and 62% secondary data sets of the studies reviewed), typically 50–90% of the data are used for training and 10–50% of the data are used for testing (Figure 4d; green circles). The *k*-fold cross validation, utilized by 3% experimental, 30% time-series, and 24% secondary data sets of the reviewed literature, is a more robust strategy (Figure 4d; green circles).^727^ Here, the data are randomly split into *k* (typically 5 or 10) folds of equal size, where *k* – 1 folds are used for training, and the remaining fold is used for testing over the *k* combinations. Finally, the average performance metrics of “*k* training” and “*k* testing” are compared to indicate whether the model overfitted or not.^728^ A variation of the *k*-fold cross validation is the “leave-one-out” cross validation, where each data sample is set aside for testing and the remaining data samples are used for training. Final performance is then obtained by taking the average over the test results from all the *N* samples. Thus, this is essentially the same as *k*-fold cross validation with *k* = *N*, where *N* is the number of data samples. The leave-one-out approach is often adopted when the number of data samples is very low, although this approach was not observed in the RRCC literature. A third, less-common strategy (7% secondary data sets of the studies reviewed) is to use primary experimental or secondary data for training and testing (Figure 4d; green circles). Notably, 73% of the studies using primary or secondary data for developing data-driven models did not split their data for estimating overfitting, likely because the purpose of majority of these models (62%) was conducting process optimization (Figure 4d; green circles). Future studies should conduct robust training and testing of data-driven models to obtain accurate models. For studies with small data sets, the leave-one-out approach is recommended.

#### 4.2.3 Feature Selection and Ranking

##### 4.2.3.1 Dimensionality Reduction and Feature Selection

An important step toward developing a data-driven model is the dimensionality reduction and feature selection.^23^ This step in data-driven modeling helps reduce redundant input variables by identifying the important drivers of the process.^729^ This step not only improves the accuracy of predictive modeling by reducing colinearity among the input variables but also improves computational efficiency.^730,731^ Dimensionality reduction is a common practice followed by data scientists during model development with high-dimensional data sets.^732,733^ Among the data-driven modeling studies that were reviewed, a considerable number of studies (86% experimental data sets, 67% time-series data sets, and 49% secondary data sets) did not include this step (Figure 4e; red circles). This discrepancy in the reviewed literature could be attributed to the current limited training and knowledge of environmental engineers in applying data science tools utilizing standard practices followed by data scientists.^23,734^ The limited studies that followed this step for developing data-driven models using primary data sets applied Pearson’s Correlation (3% experimental and 8% time-series) or Principal Component Analysis (4% experimental and 17% time-series) or Stepwise Regression (7% experimental and 8% time-series) (Figure 4e; red circles). Pearson’s Correlation is determined by measuring the direct linear relationship between two features. Although this technique is useful to identify the pairwise relationships between features, it is not helpful in reducing dimensions in the multi-dimensional space. On the contrary, Principal Component Analysis is a technique where a group of potentially correlated features are orthogonally transformed into uncorrelated groups called principal components, which helps assess the similarities and differences of the various features in high-dimensional space.^735,736^ Stepwise regression is the step-by-step process of adding or removing features into a data-driven model until the combination of features with the best performance is reached.^737^ Pearson’s Correlation was utilized most frequently (29% of the cases) by the studies developing data-driven RRCC models with secondary data sets followed by Principal Component Analysis (9%), and Stepwise Regression (9%) (Figure 4e; red circles). Spearman’s Correlation, describing the pairwise monotonic relationships between features, was infrequently applied in the RRCC literature (only 4% of the secondary data sets), a surprise considering that the processes related to the technologies are complex and monotonic (Figure 4e; red circles).^738,739^ Overall, future researchers should consider dimensionality reduction, particularly the application of Spearman’s Correlation and Principal Component Analysis, for feature selection as an essential step toward developing data-driven models of RRCC. More advanced approaches, such as using latent features from different types of autoencoders, should be considered.^740^

##### 4.2.3.2 Feature Importance Ranking

Unlike the dimensionality reduction and feature selection step where relevant features are preselected before model training, the feature importance ranking step calculates the ranking of each feature with respect to the feature’s contribution in the ML decision or prediction, and hence can be used toward explaining the trained model.^741^ Similar to the dimensionality reduction and feature selection step, feature importance ranking was also followed less in the reviewed RRCC literature for developing data-driven models; 95% experimental data sets, 83% time-series data sets, and 47% secondary data sets were used to develop models without feature importance ranking (Figure 4f; purple circles). Among statistical methods, Variable Importance in Projection was used in the limited studies that applied PLSR in RRCC (2% of experimental and 2% secondary data sets) (Figure 4f; purple circles). This method utilizes the variance explained by each of the partial least-squares component (similar to the principal components in Principal Component Analysis) to calculate scores representing the importance of each feature.^742^ Among ML methods, although ANN models inherently lack the ability to calculate feature importance, a few RRCC studies (2% experimental, 17% time-series, and 7% secondary data sets) utilized the Garson’s method of partitioning neural network weights for separately calculating the relative importance of the features (Figure 4f; purple circles).^743^ However, unlike ANN models, tree-based ML methods such as RT, RFR, and XGBoost are embedded with relative importance of features determined using the predictive accuracy of the models in the branching process developed with the different features.^744^ Such convenience could be why the reviewed studies in the RRCC literature that developed tree-based ML models (36% secondary data sets) followed the feature importance ranking step (Figure 4f; purple circles). One feature importance method that has gained in popularity since 2020 in data-driven modeling of RRCC (9% secondary data sets) is the Shapley Additive Explanation (or more commonly known as SHAP) for its generalized applicability across different ML methods (e.g., ANN, SVR, and RFR) and enhanced interpretability (i.e., not only relative importance but also positive and negative effects of the features) (Figure 4f; purple circles).^745^ SHAP utilizes game theory to compute the contribution of each feature (i.e., Shapley values) toward the prediction of a ML model.^67^ Overall, considering the availability of multiple methods for feature importance ranking (particularly robust methods like SHAP), future researchers have the opportunity to develop predictive RRCC models that are not only accurate but also interpretable.

##### 4.2.3.3 Considering Input Variables via Popularity Ranking

Identifying popular variables used in the literature could inform researchers who plan to apply data-driven methods for modeling a RRCC technology under unknown conditions. The input variables with “high” popularity (as classified in Section 2.2) can be used to develop models for generalized applications, whereas the variables with “moderate” and “low” popularity are suitable for more specific applications. Based on the applications of the different statistical and ML methods for modeling anaerobic digestion processes in the RRCC literature, the most popular input variables were operational parameters (temperature and residence time) along with feedstock properties (pH and feedstock quantities) (Table 2). The input variables to predict recovery of nutrients from liquid digestate through struvite precipitation were pH and molar ratios relevant to the chemical process (e.g., Ca^2+^/PO_4_^3–^, Mg^2+^/PO_4_^3–^, and NH_4_^+^/PO_4_^3–^), and through ammonia stripping were temperature, pH, and NH_4_^+^-N load ratio. To predict the nutrient content in composted solid digestate, C/N ratio and extractable compounds in water were used as the input variables. The C/N ratio along with other feedstock properties (pH and electrical conductivity), feedstock quantity, and composting temperature were the most popular input variables in the literature to model the nutrient content in composted feedstocks. Temperature and residence time had the most popularity for hydrothermal treatment models that were developed to predict hydrochar and biocrude yield either from solid digestates or directly from feedstocks. For the data-driven models of pyrolyzing feedstocks, operational parameters (temperature and residence time) were the most popular input variables to predict yield of biochar and bio-oil. Although no data-driven modeling studies on the pyrolysis of solid digestates were found, future studies can utilize the operational parameters to develop such models. Unlike the other thermochemical conversion methods, the most popular input variables for the gasification models to predict syngas yield were operational parameters (temperature and equivalence ratio) and feedstock properties (C–H–O–N content). Similar to the recommendations for future pyrolysis modeling studies, these input variables should be tested to develop gasification models to acquire syngas from solid digestates.

**Table 2:** Generalized Popularity of Input Variables Used in Data-Driven Models to Obtain Yields of RRCC as Outputs^a^

technology	output	popularity	input variables
Anaerobic digestion	Biogas or methane yield	High	Temperature, time, pH, and feedstock quantity
		Moderate	Volatile solids, total solids, and lignin
		Low	Loading rate, solid/water ratio, chemical oxygen demand, alkalinity, ammoniacal N, C/N ratio, lignin, cellulose, hemicellulose, lipid, protein, carbohydrates, extractives content, acid detergent fiber, catalyst, and pretreatment effect
Struvite precipitation of liquid digestate	N and P recovery	High	pH and molar ratio of calcium, magnesium, or ammonium ion to phosphate ion
Ammonia stripping of liquid digestate	Ammonia removal	High	Temperature, pH, and ammonium N load ratio
Composting of solid digestate	N and P content	High	C/N ratio and extractable compounds in water
Hydrothermal treatment of solid digestate	Hydrochar or biocrude yield	High	Temperature and time
		Moderate	pH and solvent/feedstock ratio
Composing	N and P content	High	C/N ratio, pH, electrical conductivity, temperature, and feedstock quantity
		Moderate	Time, moisture content, enzyme, dry matter, and ratio ammonium to nitrate ion
Gasification	Syngas yield	High	Temperature, equivalence ratio, and C–H–O–N content
		Moderate	Steam or calcium oxide/feedstock ratio, ash, and moisture content
		Low	Time, loading rate, particle size, fuel, or air flow rate, blending ratio, pressure, feedstock type and quantity, volatile matter, fixed C, solid content, and catalyst effect
Pyrolysis	Biochar or bio-oil yield	High	Temperature and time
		Moderate	Particle size, heating rate, and flow rate of N
		Low	Microwave power, loading rate, pressure, heating source, C–H–O–N content, ash and moisture content, volatile matter, fixed C, feedstock quantity and type, lignin, cellulose, hemicellulose, and catalyst effect
Hydrothermal treatment	Hydrochar or biocrude yield	High	Temperature and time
		Moderate	Feedstock/water ratio, C–H–O–N content, and ash content
		Low	Loading rate, heating rate, pressure, volatile matter, fixed C, moisture content, solid content, feedstock quantity, lignin, cellulose, hemicellulose, lipid, protein, carbohydrates, and catalyst effect

a Note: The method used to decide this popularity is described in Section 2.2. N = nitrogen, P = phosphorus, C = carbon, H = hydrogen, and O = oxygen.

#### 4.2.4 Performance Measurement

##### 4.2.4.1 Performance Evaluation

Performance of data-driven models is most reported in the RRCC literature as the coefficient of determination (*R*^2^) due to its easy interpretability (unitless metric with a fixed range) (Figure 4g; brown circles). Additionally, many software packages used to develop data-driven models compute *R*^2^ by default as the primary measure of predictive performance.^746,747^*R*^2^ can generally be interpreted as the proportion of the total variance about its mean explained by a model and typically ranges between 0 and 1 (i.e., *R*^2^ = 1 refers to a model that perfectly captures all variance with the input variables, and *R*^2^ = 0 indicates the model does not capture any variance).^748,749^ However, this interpretation is more appropriate for linear models than nonlinear models because additive decomposition of sum of squares and the assumption of normally distributed error do not hold during nonlinear regression.^750−752^ As a result, *R*^2^ for nonlinear models can have negative values, which indicates that the mean of the data represents a better prediction than that of the developed model.^751,753^ Additionally, the *R*^2^ measure of prediction quality suffers when errors are not normally distributed.^754^ Studies dealing with modeling nonlinear systems refer to *R*^2^ as “Pseudo-*R*^2^” in biology and “Nash-Sutcliffe efficiency” in hydrology to avoid its general interpretation.^751,755^ These studies rather interpret *R*^2^ for nonlinear models as the proportion of total variation that is not within the mean squared error of the model.^756^ Therefore, future researchers should be cautious while using *R*^2^ for evaluating performance of linear versus nonlinear models.

A majority of the reviewed RRCC studies that used experimental data sets for data-driven modeling (68%) focusing on optimization used only *R*^2^ for evaluating model performance (Figure 4g; brown circles). However, according to past studies, *R*^2^ can be unreliable when the model is developed using a small sample of data, particularly if the sample size is close to or less than 50.^757−759^ Therefore, considering the small sample size of the experimental data sets (*N* = 13–38), additional performance metrics should have been utilized for proper performance evaluation of these models. Contrary to experimental data sets, studies focused on developing predictive models with time-series and secondary data sets frequently (80% and 62%, respectively) used *R*^2^ and mean squared error (MSE) and/or root mean squared error (RMSE) for model performance evaluation (Figure 4g; brown circles). MSE represents how close predicted values are to the actual values by computing the average squared difference between the predicted and actual values, whereas RMSE is the square root of MSE for evaluating model performance in the original unit of the data set. Because errors are squared, MSE and RMSE are sensitive to extreme values in the data set, which helps indicate larger errors made by the prediction that are not captured by *R*^2^. Therefore, utilizing multiple metrics such as *R*^2^ and MSE and/or RMSE can be a useful measure for effective model performance evaluation.^760,761^ Another performance metric that was used relatively infrequently in the RRCC literature (10% primary and 20% secondary data sets) in addition to *R*^2^ and MSE and/or RMSE was mean absolute error (MAE) (Figure 4g; brown circles). The literature was inconclusive on whether to use RMSE or MAE for evaluating model performance, although the arguments ultimately rely on the variations of normal distribution of the model error.^762−764^ From that perspective, one metric that has the potential to provide a rather intuitive measure of performance for nonlinear models is the Akaike Information Criterion (AIC), which penalizes models for the higher number of parameters and not for the lack of normally distributed error.^747,765^ Surprisingly, only one of the studies reviewed in the RRCC literature utilized AIC to evaluate the accuracy of the developed ML models.^304^ Therefore, we recommend that future RRCC studies focusing on data-driven modeling should compare AIC in addition to *R*^2^, RMSE, and MAE for a more comprehensive and accurate evaluation of nonlinear models.

##### 4.2.4.2 Overfitting Estimation

The reviewed RRCC studies that split data into training and testing sets in the data pre-processing step (e.g., Single Hold-out Approach, *k*-fold Cross validation) estimated overfitting of their developed models by comparing the performance metrics (i.e., *R*^2^, *R*^2^ and MSE and/or RMSE). Overfitting occurs when a model performs well for the training set but performs poorly for the testing set (discussed in Section 4.2.2.3). Past reviews in data-driven modeling of anaerobic digestion have attributed small sample data to model overfitting because such data lack sufficient information for developing a robust predictive model.^42^ Based on the findings of the reviewed RRCC literature, the training and testing *R*^2^ values of the different statistical and ML methods applied for the prediction of RRCC technologies was compared to illustrate examples of estimating overfitting (Figure 4h; pink box and whisker plots). In this comparison, we included only the nonlinear models considering the difference of interpreting *R*^2^ for linear versus nonlinear models in addition to the ability of these models to predict the highly complex chemical and biological processes involved in the RRCC technology over linear models (discussed in Section 4.2.4.1). The median training and testing *R*^2^ values of the various data-driven methods were generally aligned (training *R*^2^ = 0.80–0.99 and testing *R*^2^ = 0.82–0.97), although the ranges of training *R*^2^ values (0.64–1.00) were slightly larger than the testing *R*^2^ values (0.54–0.99) (Figure 4h; pink box and whisker plots). The overall comparison, therefore, indicated that the studies reviewed in the literature that conducted data splitting (more than 90% of time-series and secondary data sets but less than 30% of experimental data sets) effectively estimated overfitting of their developed models. Overfitting of models could be avoided by assessing various cross-validation techniques for a particular data set (discussed in Section 4.2.2.3). Overfitting can also be contributed by data leakage, where testing data leaks into the training data set and/or irrelevant features leak into model training, thereby affecting the predictive ability of the model. To avoid data leakage and subsequent overfitting, data pre-processing procedures can be conducted separately for each cross-validation split instead of conducting the procedures collectively. Further, conducting dimensionality reduction and feature selection on the entire data set instead of splitting can help minimize data leakage. Overall, it is our strong recommendation for future research on data-driven modeling of RRCC to place a greater emphasis on estimating overfitting considering the use of developed models by other researchers utilizing unknown data sets.

### 4.3 Important Considerations for Applying Data Science Methods in Environmental and Economic Impact Analyses

**Figure 5:**
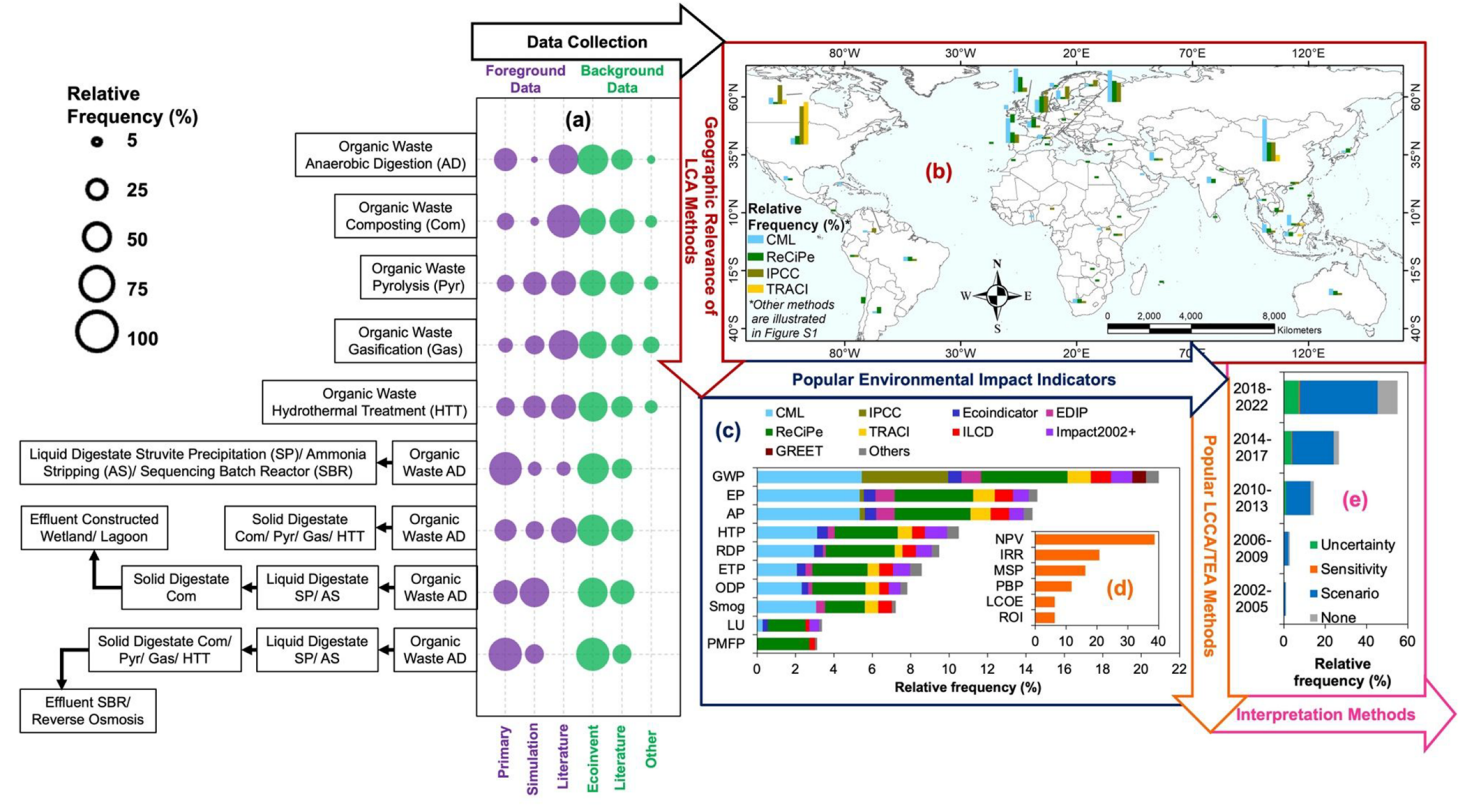
Summary of key considerations for applying data science methods for the environmental and economic impact analyses of RRCC technologies at the different stages of model development: (a) relative frequency of foreground (purple circles) and background (green circles) systems data for collection with respect to different treatment processes, (b) geographic relevance of life cycle assessment (LCA) methods (relative frequencies of methods within the map with red border), (c) popular environmental impact indicators (relative frequencies of indicators within the bar chart with blue border), (d) popular life cycle cost analysis/techno-economic analysis (LCCA/TEA) methods for economic impacts (relative frequencies of indicators with orange bar chart), and (e) methods for interpreting the LCA and LCCA/TEA indicators (relative frequencies of methods within the bar chart with pink border). (Notations: GWP = Global Warming Potential, EP = Eutrophication Potential, AP = Acidification Potential, HTP = Human Toxicity Potential, RDP = Resource Depletion Potential, ETP = Eco-Toxicity Potential, ODP = Ozone Depletion Potential, LU = Land Use, PMFP = Particlate Matter Formation Potential, NPV = Net Present Value, IRR = Internal Rate of Return, MSP = Minimum Selling Price, PBP = Payback Period, LCOE = Levelized Cost of Energy, ROI = Return on Investment)

Based on our review of the literature, the following aspects are important to consider while applying data science methods for the environmental and economic impact analyses of RRCC technologies at the different stages of model development (Figure 5).

#### 4.3.1 Data Collection

Generally, data from two types of systems were utilized for conducting environmental and economic impact analyses applying the LCA and LCCA/TEA methods: foreground systems and background systems (Figure 5a; purple and green circles, respectively). Foreground systems refer to all the processes specific to the RRCC technology (e.g., percent yield of recovered resources under specific organic waste composition and operational conditions like temperature and residence time) along with the processes that can directly affect the decisions of the study (e.g., area required for centralized operation or transportation distance for decentralized operation).^328,766^ Background systems, on the other hand, refer to the processes that are influenced by the foreground systems (e.g., amount of greenhouse gases emitted from the electricity use for operating a technology under specific conditions). Among the reviewed literature, studies that focused on implementing a single RRCC technology (anaerobic digestion, composting, pyrolysis, gasification, or hydrothermal treatment) utilized past literature data relatively more for foreground systems (42–75%) than primary (15–37%) or simulation data (3–37%), probably because such implementation was more common in the literature (407 cases) than integrated implementation of RRCC technologies (64 cases) (Figure 5a; purple circles). Studies with integrated implementation of RRCC technologies that focused on treating solid digestate from anaerobic digestion via composting, pyrolysis, gasification, or hydrothermal treatment used relatively more literature data (45%) than primary (33%) or simulation data (23%) for the foreground systems (Figure 5a; purple circles). However, primary data for foreground systems were utilized by a majority of the integrated implementations of RRCC technologies that focused on treating liquid digestate from anaerobic digestion via struvite precipitation or ammonia stripping or sequencing batch reactor (73%) where researchers relied on their pilot-scale experiments (Figure 5a; purple circles). The limited studies that integrated anaerobic digestion with liquid and/or solid digestate treatment as well as treatment of effluent from digestate treatment (constructed wetland or lagoon or sequencing batch reactor) only used primary (40–75%) or simulation (25–60%) data for foreground systems (Figure 5a; purple circles). Contrary to the foreground data, the reviewed studies in RRCC frequently used Ecoinvent as their data source for background systems (48–75%) followed by data obtained from past literature studies (25–43%). In some other cases (5–18%), Ecoinvent was combined with data from the literature or country-specific repositories such as United States Life Cycle Inventory (Figure 5a; green circles). Overall, based on the availability of data sources, researchers have the option to utilize multiple data sources from the literature for foreground systems (if limited financial resources exist to perform real-time experiments) and can greatly rely on Ecoinvent for background systems to conduct environmental and economic impact analyses of both single and integrated approaches of RRCC technologies.

#### 4.3.2 Geographic Relevance of LCA Methods

The review of the LCA methods applied to assess the environmental impacts of RRCC technologies indicated that the different methods varied by geographical region. Based on the frequency of the different LCA methods applied in different countries, we found that the most applications were in USA followed by China, and European countries (Figure 5b; map in red border). TRACI, IPCC, and GREET were the most applied LCA methods in USA (Figure 5b; map in red border and Figure S1). In China, CML was applied most frequently followed by EDIP, IPCC, and ReCiPe (Figure 5b; map in red border and Figure S1). CML, ReCiPe, and IPCC were among the common LCA methods that were applied in other Asian countries, namely India, Singapore, Malaysia, and Iran (Figure 5b; map in red border). In Europe, all the LCA applications were in the western region (particularly Italy, Spain, United Kingdom, Germany, Belgium, and Sweden) where CML, ReCiPe, and IPCC were the most frequently applied methods (Figure 5b; map in red border). EDIP was most applied for LCA studies in Denmark after China, which is expected because this method was developed by the Danish Environmental Protection Agency (Figure S1).^91^ The applications of TRACI in LCA studies in the USA is understandable considering that it was developed by the United States Environmental Protection Agency.^94^ The older CML method and the much newer ReCiPe method have been more generally applied around the world to conduct LCA of different RRCC technologies. The frequent applications of IPCC were mostly based on the most updated characterization factors of global warming potential, and in many cases, IPCC was combined with other methods. Overall, CML and ReCiPe were the most popular methods on a global scale, which is consistent with the findings of the systematic review by Mulya et al.^767^ However, our study provides the additional insight that in terms of regional scale of LCA methods, TRACI is representative of the USA, and ILCD is representative of the western European countries.

#### 4.3.3 Popular Environmental Impact Indicators and LCCA/TEA Methods for Economic Impact Analysis

Among the different environmental impact indicators quantified for the LCA of RRCC technologies, global warming potential was reported most frequently in the literature followed by eutrophication and acidification potential (Figure 5b; bar chart in blue border). One of the key purposes of introducing RRCC technologies is to mitigate the adverse impacts of climate change caused by conventional organic waste disposal practices (e.g., greenhouse gas emissions from landfilling). Additional impacts of such waste disposal practices include the release of toxins and leachate from landfilling, along with harmful byproducts from incineration into the environment that can eventually cause eutrophication and acidification.^768^ Therefore, it is understandable that these three environmental impact indicators were the most reported in the LCA studies. Among other environmental impact indicators, human toxicity, ecotoxicity, resource depletion, ozone depletion, and smog potential were reported relatively less in the RRCC literature. For LCCA/TEA, the most frequently applied method was NPV followed by IRR, MSP, and PBP (Figure 5c; bar chart in with orange bars).

#### 4.3.4 Methods for Interpreting the Quantified Impacts from LCA and LCCA/TEA

The use of different interpretation methods of the environmental and economic indicators increased over the past decade (Figure 5e; bar chart in pink border). Scenario analysis was applied most frequently during 2002–2022. For uncertainty analysis, the use of Monte Carlo simulation has increased since 2010. Monte Carlo simulation refers to an empirical process of repeated sampling (typically 10,000 repetitions in the reviewed RRCC literature) to investigate the uncertain behavior of a data-intensive complex system.^769^ Among the limited implementation of sensitivity analysis, Pearson’s Correlation and Pedigree matrix were commonly utilized since 2016. Pearson’s Correlation is a quantitative technique for assessing the sensitivities of the drivers of the environmental and economic impacts of a RRCC technology (discussed in Section 4.2.3.1). On the contrary, Pedigree matrix is a rather qualitative approach for assessing such sensitivities that is based on reliability, completeness, temporal, geographic, and technological representativeness.^770^ Spearman’s Correlation (discussed in Section 4.2.3.1) can also be considered as quantitative technique for such sensitivity analysis.^771^ Therefore, this survey of the RRCC literature reveals a lack of sensitivity analysis for LCA and LCCA/TEA indicators. Future work may consider the incorporation of uncertainty and sensitivity analyses to aid in developing a more comprehensive understanding of the drivers of the environmental and economic impacts.

## 5 Toward an Integrated Data Science Approach to Inform Sustainable RRCC from Organic Waste

Data-driven modeling tools such as ML methods can often leverage real-time monitoring sensor data in modern RRCC infrastructure to improve the efficiency of treatment processes or provide additional benefits over conventional practices where process controls are informed by past performance and operator’s experience.^23,772−774^ However, at the current global climate where authorities are pushing waste management infrastructures to adopt practices for reducing environmental impacts while maintaining economic prosperity, the priority for achieving process efficiency for RRCC has developed.^775,776^ For example, at the end of 2022, United States Department of Energy issued a USD 23 million funding opportunity to drive innovative decarbonization strategies to reduce greenhouse gas emissions from public water resource recovery infrastructure in the USA by 25% without increasing the overall cost.^777^ Although these infrastructures have achieved high RRCC efficiency over the past decade, large amounts of energy are consumed by the underlying treatment processes that are resulting in life cycle emissions close to that of the cement industry (approximately 44 t of CO_2_ equivalents).^778−780^ One possible approach that can aid the research and development of such innovative decarbonization strategies could be the integration of data-driven models for process modeling with LCA and LCCA/TEA for the environmental and economic impact analyses, respectively.

Our review indicates a need for integrating data-driven modeling with LCA and LCCA/TEA in the RRCC literature (two pyrolysis studies and one hydrothermal treatment study discussed in Section 3.2.4 and 3.2.6, respectively). One reason why the literature lacks such integration of data science methods could be that environmental engineers generally have limited training about the applications of data-driven modeling and lack adequate skills to facilitate such integration through scientific programming.^23,734^ A recent survey of civil and environmental engineering departments in more than 40 renowned universities in the USA demonstrated that only 7% provided training in data science tools and scientific programming in popular languages like Python and R to undergraduate students.^781^ The survey, however, revealed that 85% of these departments believed the importance of developing such skills for the next generation of environmental engineers considering the emergence of big data. Additionally, many environmental engineering researchers who do not have access to RRCC infrastructure data are faced with data availability issues while working on developing data-driven modeling solutions (discussed in Section 4.2.1). Unlike the established data sets to conduct LCA and LCCA/TEA of RRCC technologies such as Ecoinvent and United States Life Cycle Inventory,^105,782^ large data sets are still lacking in the data-driven modeling domain, although more recent publications show significant improvement in the availability of more and increasingly larger data sets (discussed in Section 4.2.1). Overall, considering the abundance of data in modern RRCC facilities and the urgent need for their sustainable and efficient operations, it is our recommendation to establish a national or global open-source repository of quality-controlled time-series data sets (e.g., AmeriFlux or FLUXNET in ecosystem modeling)^783,784^ from RRCC facilities that can be readily accessible for environmental engineering researchers to work on formulating and testing innovative strategies for implementing RRCC utilizing data science tools.

**Figure 6:**
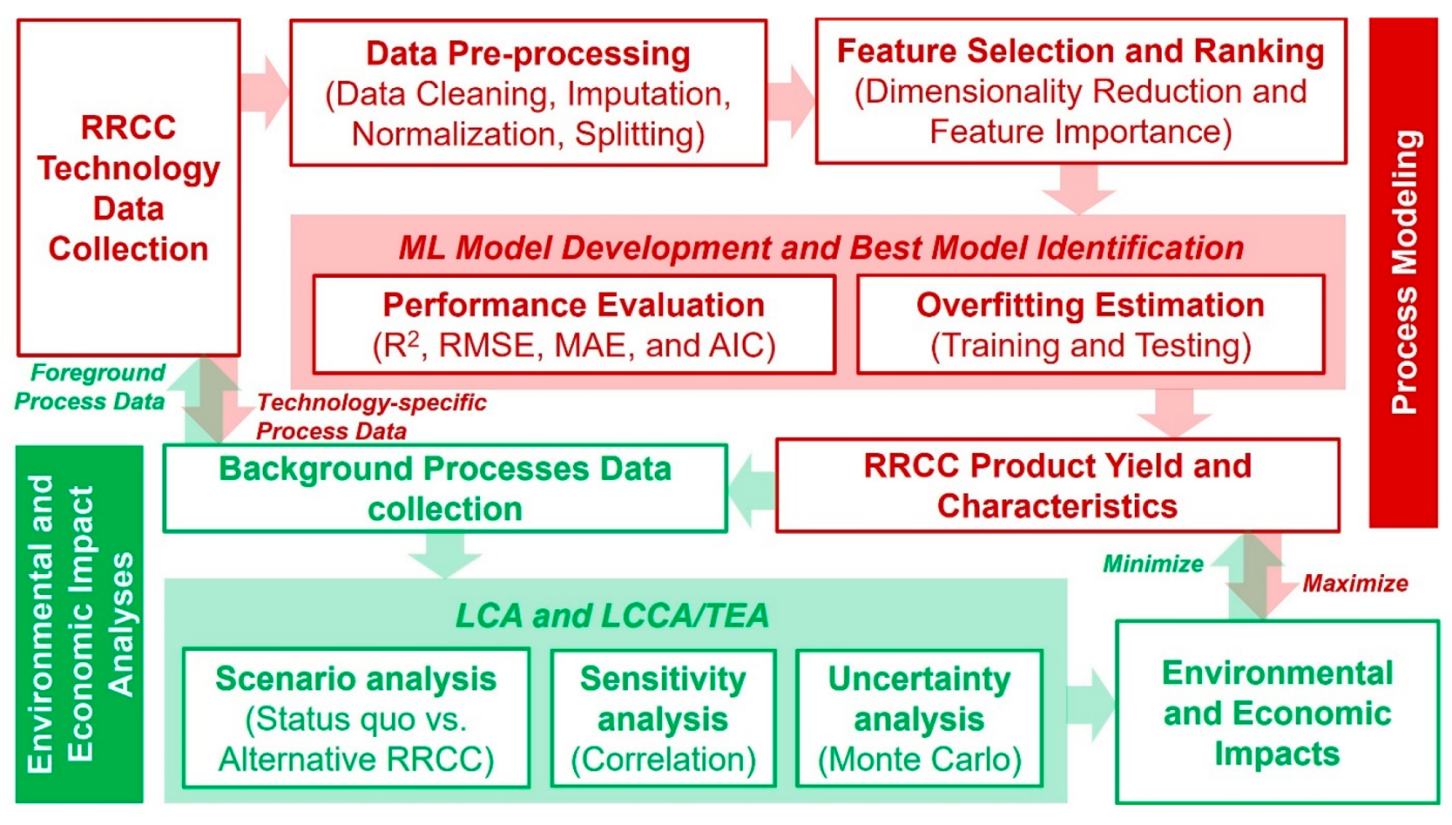
Proposed framework to inform integrated, data-driven sustainable design of RRCC from organic waste streams.

Therefore, we propose a framework that can work as a systematic path for integrating ML methods with LCA and LCCA/TEA to inform efficient and sustainable RRCC from organic waste based on the review of 616 peer-reviewed articles published during 2002–2022 (Figure 6), as discussed in Section 4. The framework has two stages: a process modeling stage and an environmental and economic impact analyses stage as described in the following two paragraphs, respectively.

At the process modeling stage of the framework, the collected RRCC technology-specific data from multiple sources are combined to create a secondary data set through data blending as opposed to a primary data set where the data is likely collected from the real-time monitoring sensors of an RRCC infrastructure (details in Section 4.2.1). The collected data are then pre-processed through cleaning and imputation (if necessary, after visual investigation of the data) followed by the necessary data normalization and splitting (details in Section 4.2.2). Next, dimensionality reduction and feature importance methods can be applied to identify important input variables and select the key features to model the processes of the RRCC technology (details in Section 4.2.3). Utilizing the important input variables (or key features), ML models can be evaluated with respect to training and testing performances using *R*^2^, RMSE, MAE, and AIC (details in Section 4.2.4). At the final step of the process modeling stage, the output of the best ML model representing the RRCC product yields and characteristics (i.e., efficiency of the treatment process such as biogas yield, nutrient recovery, biochar energy content) can be fed into LCA and LCCA/TEA models.

At the environmental and economic impact analyses stage of the framework, the LCA and LCCA/TEA of the treatment process are conducted using foreground and background process data (details in Section 4.3.1). One crucial consideration while collecting the background process data for LCA and LCCA/TEA is that it must be complemented by the technology-specific process data through the foreground processes. For example, studies that performed such integration of data science methods in the past utilized ML models for running the foreground processes by predicting yield and energy content of recovered resources using operational conditions (e.g., temperature) and then connecting those outputs with background systems (e.g., heat consumption and offset using predicted energy content) to conduct LCA and LCCA/TEA. Based on the trends of past studies, ReCiPe can be used globally and TRACI in the USA to conduct an LCA (details in Section 4.3.2) by quantifying the global warming, eutrophication, and acidification potential, whereas NPV can be applied for LCCA/TEA (details in Section 4.3.3). An important final step to interpret the results of the LCA and LCCA/TEA is conducting scenario, sensitivity, and uncertainty analyses (details in Section 4.3.4). Overall, this framework can be used by environmental engineers as a stepping stone for future research related to investigating the trade-off between efficient and sustainable RRCC from organic waste streams.

The proposed integration of data science tools is important for cost-effective implementation of RRCC at full scale, given the potential of data-driven models for effectively scaling up technology processes with the applications of LCA and LCCA/TEA to assess their environmental-economic impacts in a virtual environment.^24,785^ The use of such an integrated approach can benefit biorefineries in the USA that are struggling to operate at an industrial production level. The United States Department of Energy and private industries recently invested more than $2 billion on extensive research and development to scale-up these operations.^786^ Further, utilizing commercial process simulation tools for such purposes are considered costly, computationally expensive, and lack reproducibility, which can be overcome using ML integrated LCA and LCCA/TEA.^787^ Additionally, although not in the scope of this study, it may also be beneficial to consider quantifying social acceptabilities of implementing RRCC, particularly in urban contexts.^371,458,593,678^ One study assessed the social impacts of implementing anaerobic digestion in an animal farm in a rural context through a stakeholder perception study.^475^ Apart from such qualitative approaches, a quantitative approach can be the Guidelines for Social Life Cycle Assessment of Products.^788^ The Guidelines for Social Life Cycle Assessment of Products approach assesses the social impacts in terms of social responsibility across seven aspects: institutional domination, human rights, labor affairs, environment, fair work, consumer rights, and participation in local development. This has been utilized to assess the social impacts of anaerobically digesting animal waste.^789^ Therefore, in addition to integrating data-driven modeling for cost-effective implementations, it is essential to combine social impact assessments with the rising applications of LCA and LCCA/TEA to achieve environmental, economic, and social sustainability of implementing RRCC technologies both in rural and urban settings.

## 6 Conclusion

This critical review indicates that the use of data science in RRCC is largely unexplored in terms of integrated approaches for multiple technologies with the potential to utilize different feedstocks and recover various resources. Applications of ML methods for process modeling in RRCC has drastically increased over time where the tree-based algorithms were most popular to model the technologies. Of the literature reviewed, limited studies utilized optimization algorithms within the ML models such as particle swarm optimization^790^ and genetic algorithm.^791^ These algorithms have been employed either to optimize the hyperparameters of the ML models (i.e., number of hidden layers in ANN, number of trees in RFR)^227,232,267,306^ or the input variables such as operational parameters and feedstock properties to attain maximum output for RRCC (i.e., biogas yield or biochar yield).^158,162,165,171,193,243^ Incorporating such optimization algorithms with ML methods can further improve the efficiency of real-time modeling of RRCC processes. In terms of sustainability, an increasing number of studies are including LCCA/TEA with LCA to quantify both environmental and economic impacts of RRCC. Globally, ReCiPe and CML were the most used LCA methods for environmental impact analysis in RRCC, although TRACI was commonly leveraged in the USA and ILCD in European countries. NPV was the most used method for LCCA/TEA. For a more comprehensive sustainability assessment of the RRCC technologies, future research may integrate social impact assessment methods such as Guidelines for Social Life Cycle Assessment of Products with these LCA and LCCA/TEA methods.

Overall, in terms of data science, the literature lacked integration of statistical or ML methods for process modeling with LCA and LCCA/TEA for the environmental and economic impact analysis of the different RRCC technologies. However, the lack of such studies in the literature can be fulfilled by developing open-source packages by integrating already existing ML tools in Python (e.g., Sci-kit learn)^792^ with the LCA and LCCA/TEA methods following our proposed framework. Open-source Python packages for LCA and LCCA/TEA such as QSDSan (Quantitative Sustainable Design of Sanitation and Resource Recovery Systems),^771,793^ SwolfPy (Solid waste optimization life-cycle framework in Python)^794^ and WaterTAP^795^ may provide a foundation to develop such integrated tools. A major advantage of these packages is built-in uncertainty and sensitivity analyses methods. Using the data science methods recommended by this study (e.g., RFR for process modeling and ReCiPe and NPV for LCA and LCCA/TEA, respectively) and with the appropriate open-source tools, future researchers can simulate RRCC technologies to help inform the sustainable design of waste management infrastructure. Currently, many utilities in larger urban areas collect data sets that could inform RRCC using the methods.^36^ These methods also have the potential to inform synergistic opportunities between rural communities (that lack financial resources) and nearby agricultural industries. Overall, data science can be leveraged to achieve global sustainability goals around RRCC by helping to identify opportunities for advanced technologies and optimize their efficiencies.

## Supporting Information Available

Additional details on past literature review articles (Table S1), literature search in Google Scholar (Table S2), life cycle assessment (LCA) methods applied in the reviewed literature (Table S3), peer-reviewed journals in the reviewed literature focused data-driven modeling (Table S6) and LCA and life cycle cost analysis/techno-economic analysis (LCCA/TEA) (Table S7), frequency analysis of input variables in data-driven modeling (Table S8), frequency of applications in data-driven modeling (Table S9) and LCA and LCCA/TEA in reviewed literature (Table S10), and geographic relevance of LCA applications in reviewed literature (Figure S1) (PDF).
Detailed records of the collected information from reviewed literature of data-driven modeling (Table S4) and LCA and LCCA/TEA (Table S5) (XLSX).
