# Supplementary material for: A Critical Review of Data Science Applications in Resource Recovery and Carbon Capture from Organic Waste
Source: ACS ES T Eng. 2023 Sep 29;3(10):1424–67. doi: 10.1021/acsestengg.3c00043 (PMC10580293; doi:10.1021/acsestengg.3c00043)
Supplement: Supplementary file 1 — ee3c00043_si_001.pdf [file ee3c00043_si_001.pdf]

A Critical Review of Data Science Applications

in Resource Recovery and Carbon Capture from Organic Waste

Mohammed T. Zaki[a], Lewis S. Rowles[b], Donald A. Adjeroh[c], and Kevin D. Orner[a,*]

Affiliations: [a]Wadsworth Department of Civil and Environmental Engineering, West Virginia University, Morgantown, WV 26505, USA; [b]Department of Civil Engineering and Construction, Georgia Southern University, Statesboro, GA 30458, USA; [c]Lane Department of Computer Science and Electrical Engineering, West Virginia University, Morgantown, WV26505, USA

*Corresponding author: kevin.orner@mail.wvu.edu

Supplemental Information

List of contents

Table S1. Summary of findings of existing review articles on the applications of data science in resource recovery and carbon capture.

Table S2. Keywords used in Google Scholar for the literature search.

Table S3. List of various life cycle assessment methodologies.

Table S4. Records of the relevant information from literature studies on the applications of data-driven methods for process modeling in resource recovery and carbon capture from organic waste streams.

Table S5. Records of the relevant information from literature studies on the applications of environmental and economic impact analysis tools in resource recovery and carbon capture from organic waste streams.

Table S6. Number of data-driven modeling studies in resource recovery and carbon capture published in the different peer-reviewed journals.

Table S7. Number of LCA, LCCA, and TEA studies in resource recovery and carbon capture published in the different peer-reviewed journals during 2002-2022.

Table S8. Frequency (f) and relative frequency (Rf) of the input variables used for developing data-driven models for resource recovery and carbon capture technologies (Tech) in literature during 2002-2022.

Table S9. Percentage (%) of different data-driven modeling applications in resource recovery and carbon capture literature.

Table S10. Percentage (%) of different environmental and economic analysis applications in resource recovery and carbon capture literature.

Figure S1. Number of applications of the various life cycle assessment methods with respect to the different countries around the world in resource recovery and carbon capture from organic waste literature.

Table S1. Summary of findings of existing review articles on the applications of data science in resource recovery and carbon capture. Note that reference numbers from the main text.

Organic waste stream	Technology	Recovered resource	Data Science tool	Reference
Agricultural crop and forestry residue	Pyrolysis	Biofuel	Statistical and ML methods	[37]
Organic municipal solid waste	Anaerobic digestion	Biogas	ML methods	[38]
Agricultural crop residue, organic municipal solid waste and sewage sludge, and animal manure	Anaerobic digestion	Biogas	ML methods	[39]
Agricultural crop residue, organic municipal solid waste and sewage sludge, and animal manure	Gasification and hydrothermal treatment	Biofuel	ML methods	[40]
Agricultural crop residue, organic municipal solid waste and sewage sludge, and animal manure	Gasification and pyrolysis	Biochar and biofuel	ML methods	[24]
Agricultural crop residue and animal manure	Anaerobic digestion	Biogas	LCA	[41]
Agricultural crop residue, organic municipal solid waste and sewage sludge, and animal manure	Anaerobic digestion	Biogas	LCA and TEA	[42]
Organic municipal solid waste	Anaerobic digestion, pyrolysis, gasification, and hydrothermal treatment	Biogas, biochar, syngas, and hydrochar	LCA	[43]
Agricultural crop residue, organic municipal solid waste and sewage sludge, and animal manure	Anaerobic digestion, pyrolysis, gasification, and hydrothermal treatment	Biogas, biochar, syngas, and hydrochar	LCA	[44]
Municipal sewage sludge	Anaerobic digestion, pyrolysis, and hydrothermal treatment	Biogas, biochar, and hydrochar	LCA	[45]
Agricultural and forestry residue	Pyrolysis	Biochar and biofuel	LCA	[46]

Note: ML = machine learning, LCA = life cycle assessment, and TEA = techno-economic analysis.

Table S2. Keywords used in Google Scholar for the literature search.

Type		Keyword
Feedstock		Agricultural, forest residue, woody biomass, municipal solid waste, animal manure, sewage sludge
Treatment technology	1st step	Anaerobic digestion, composting, pyrolysis, gasification, hydrothermal
	2nd step	Struvite, stripping, composting, pyrolysis, gasification, hydrothermal
	3rd step	Wetland, lagoon, wastewater treatment
Data science tool		Regression, machine learning, life cycle
Recovered resource		Biogas, nutrients, fertilizer, bio-oil, biochar, syngas, biocrude, hydrochar, water

Table S3. General information of various life cycle assessment methodologies typically used in RRCC literature.

Method	Developer	General environmental impact categories used in RRCC
Centrum voor Milieukunde Leiden (CML)	Leiden University, Netherlands	GWP, EP, AP, HTP, ETP, ODP, Smog, RDP, LU
Eco-Indicator	PRé Sustainability, Netherlands	GWP, EP, AP, HTP, ETP, ODP, RDP, LU
Environmental Development of Industrial Products (EDIP)	Danish Environmental Protection Agency	GWP, EP, AP, HTP, ETP, ODP, Smog, RDP
Intergovernmental Panel on Climate Change (IPCC)	IPCC	GWP, EP, AP
RIVM, CML and, PRé Consultants (ReCiPe)	RIVM, Radboud University Nijmegen, Leiden University and PRé Sustainability, Netherlands	GWP, EP, AP, HTP, ETP, ODP, Smog, PMFP, RDP, LU
Tool for the Reduction and Assessment of Chemical and Other Environmental Impacts (TRACI)	United States Environmental Protection Agency	GWP, EP, AP, HTP, ETP, ODP, Smog, RDP
International reference Life Cycle Data System (ILCD)	European Union	GWP, EP, AP, HTP, ETP, ODP, Smog, PMFP, RDP, LU
Impact 2002+/World+	Quantis (Europe and United States)	GWP, EP, AP, HTP, ETP, ODP, RDP, LU
Greenhouse gases, Regulated Emissions, and Energy use in Technologies (GREET)	Argonne National Laboratory, United States	GWP
Environmental Footprint (EF)	European Union	GWP, EP, AP, HTP, ETP, ODP, Smog, PMFP, RDP, LU
USEtox	European Union, United States, and China	HTP, ETP
Economic Input-Output LCA (EIO-LCA)	Carnegie Mellon University, United States	GWP
Environmental Load Point (ELP)	Linuhonnun Consulting, Iceland; Det Norske Veritas, Norway; The Environmental Strategies Research Group, Stockholms University; The Department of Manufacturing Engineering and Management, Technical University of Denmark	GWP, EP, AP, HTP, ETP, ODP, RDP
Environmental Product Declaration (EPD)	EPD International, Sweden	GWP, EP, AP, Smog
Global Emission Model for Integrated Systems (GEMIS)	Öko-Institut (Institute for Applied Ecology), Germany	GWP
Stepwise2006	Combination of Impact 2002+ and EDIP	GWP, EP, AP, HTP, ETP, ODP, RDP
National GHG inventory report of Japan	National Institute of Advanced Industrial Science and Technology and the Japan Environmental Management Association for Industry	GWP, EP, AP, ODP, RDP

Note: GWP = Global warming potential, EP = Eutrophication potential, AP = Acidification potential, HTP = Human toxicity potential, ETP = Eco-toxicity potential, ODP = Ozone depletion potential, PMFP = Particulate matter formation potential, RDP = Resource depletion potential, LU = Land use.

Table S4. Records of the relevant information from literature studies on the applications of data-driven methods for process modeling in resource recovery and carbon capture from organic waste streams.

Table S4 is included as a Microsoft Excel Spreadsheet: SI_DataScience-CriticalReview.xlsx

Table S5. Records of the relevant information from literature studies on the applications of environmental and economic impact analysis tools in resource recovery and carbon capture from organic waste streams.

Table S5 is included as a Microsoft Excel Spreadsheet: SI_DataScience-CriticalReview.xlsx

Table S6. Number of data-driven modeling studies in resource recovery and carbon capture published in the different peer-reviewed journals.

**Journal name**	**No.**	**Journal name**	**No.**
Bioresource Technology	29	Chemical Engineering Transactions	1
Fuel	16	Clean Technologies and Environmental Policy	1
Biomass Conversion and Biorefinery	10	Energies	1
Energy	10	Energy Exploration & Exploitation	1
Biomass and Bioenergy	9	Energy Sources, Part A: Recovery, Utilization, and Environmental Effects	1
Energy Conversion and Management	9	Engineering in Agriculture, Environment and Food	1
Applied Energy	6	Environment, Development and Sustainability	1
International Journal of Hydrogen Energy	6	Environmental Engineering Research	1
Renewable Energy	5	Fermentation	1
Fuel Processing Technology	4	Frontiers in Energy Research	1
Journal of Analytical and Applied Pyrolysis	4	Gasification for Practical Applications	1
Journal of Cleaner Production	4	In Proceedings of the Institution of Civil Engineers-Municipal Engineer	1
Waste Management	4	Industrial Crops and Products	1
BioResources	3	Information Processing in Agriculture	1
Chemical Engineering Journal	3	International Conference on Applied Energy	1
Energy & Fuels	3	International Journal of Energy and Environmental Engineering	1
International Journal of Energy Research	3	International Journal of Environmental Research and Public Health	1
Journal of Environmental Chemical Engineering	3	International Journal of Environmental Science and Technology	1
Science of the Total Environment	3	International Journal of Renewable Energy Development	1
Waste and Biomass Valorization	3	International Journal of Renewable Energy Research	1
Bioresource Technology Reports	2	IOP Conference Series: Materials Science and Engineering	1
Environmental Technology & Innovation	2	Journal of Environmental Management	1
Heliyon	2	Journal of Environmental Quality	1
Resources, Conservation and Recycling	2	Journal of Industrial and Engineering Chemistry	1
Waste Management & Research	2	Journal of Intelligent Systems	1
3 Biotech	1	Journal of King Saud University - Science	1
ACS ES&T Engineering	1	Journal of The Institution of Engineers (India): Series E	1
ACS Omega	1	Modeling Earth Systems and Environment volume	1
ACS Sustainable Chemistry & Engineering	1	Nigeria Journal of Engineering and Applied Sciences	1
African Journal of Science, Technology, Innovation and Development	1	Procedia Environmental Sciences	1
Alexandria Engineering Journal	1	Process Biochemistry	1
Applied Energy Symposium	1	Process Safety and Environmental Protection	1
Asian-Australasian journal of animal sciences	1	Renewable and Sustainable Energy Reviews	1
Biofuels	1	Resource-Efficient Technologies	1
Biomass Now-Sustainable Growth and Use	1	Scientific reports	1
BioMed Research International	1	Sustainable Energy Technologies and Assessments	1
Bioresources and Bioprocessing	1	The Global Environmental Engineers	1
Biosystems Engineering	1	The Journal of Supercritical Fluids	1
Canadian Biosystems Engineering	1	Water Research	1
Chemical Engineering and Processing-Process Intensification	1	Wood Science and Technology	1

Table S7. Number of LCA, LCCA, and TEA studies in resource recovery and carbon capture published in the different peer-reviewed journals during 2002-2022.

Journal name	No.	Journal name	No.
Journal of Cleaner Production	66	Atmospheric Environment	1
Bioresource Technology	33	BioEnergy Research	1
Waste Management	30	Biomass Conversion and Biorefinery	1
Science of the Total Environment	24	BioResources	1
Applied Energy	19	Chemical Engineering and Processing: Process Intensification	1
Environmental Science & Technology	16	Chemical Engineering Journal	1
Renewable Energy	16	Cleaner Environmental Systems	1
Resources, Conservation and Recycling	15	Detritus	1
Biomass and Bioenergy	12	Ecological Indicators	1
Energies	12	Energy Exploration & Exploitation	1
Energy Conversion and Management	12	Energy Policy	1
Journal of Environmental Management	11	Energy Procedia	1
Energy	10	Energy Technology	1
Renewable and Sustainable Energy Reviews	10	Energy, Sustainability and Society	1
ACS Sustainable Chemistry & Engineering	9	Environment International	1
The International Journal of Life Cycle Assessment	8	Environmental Engineering & Management Journal	1
Journal of Material Cycles and Waste Management	6	Environmental Engineering Science	1
Waste and Biomass Valorization	6	Environmental Pollution	1
Waste Management & Research	5	Environmental Research	1
Sustainability	4	Environmental Research Letters	1
Energy & fuels	3	Environmental Technology	1
Environmental Progress & Sustainable Energy	3	Frontiers in Bioengineering and Biotechnology	1
Environmental Science and Pollution Research	3	Global Journal of Environmental Research	1
Fuel	3	Green Chemistry	1
International Journal of Energy and Environmental Engineering	3	Heliyon	1
Water Science and Technology	3	Integrated Environmental Assessment and Management	1
Biofuels, Bioproducts and Biorefining	2	International Congress Series	1
Biotechnology for Biofuels	2	International Journal of Environmental Science and Technology	1
Chemical Engineering Transactions	2	International Journal of Green Energy	1
Chemosphere	2	International Journal of Renewable Energy Technology Research	1
Clean Technologies and Environmental Policy	2	Journal of Energy Resources Technology	1
Engineering in Life Sciences	2	Journal of Environmental Sciences	1
Environmental Monitoring and Assessment	2	Journal of Forestry Research	1
Forest Products Journal	2	Journal of Industrial Ecology	1
GCB Bioenergy	2	Journal of Natural Gas Science and Engineering	1
International Journal of Hydrogen Energy	2	Journal of Sustainable Development of Energy, Water and Environment Systems	1
Process Safety and Environmental Protection	2	Land	1
Sustainable Energy Technologies and Assessments	2	Process Integration and Optimization for Sustainability	1
Water Research	2	Progress in Life Cycle Assessment	1
Agronomy	1	Scientific Reports	1
Animal	1	Sustainable Cities and Society	1
Applied Sciences	1	Sustainable Energy & Fuels	1
Applied Thermal Engineering	1	Sustainable Production and Consumption	1
ASABE Annual International Meeting	1	Waste Disposal & Sustainable Energy	1
Asian-Australasian Journal of Animal Sciences	1	Water	1

Table S8. Frequency (f) and relative frequency (Rf) of the input variables used for developing data-driven models for resource recovery and carbon capture technologies (Tech) in literature during 2002-2022.

Tech	Input variables	f	Rf
**AD**	Feedstock quantity	29	1.00
	Temperature	21	0.72
	Time	21	0.72
	pH	17	0.59
	Volatile solids	11	0.38
	Total solids	10	0.34
	Lignin	9	0.31
	Volatile fatty acids	7	0.24
	Chemical oxygen demand	7	0.24
	Organic loading rate	6	0.21
	Catalyst effect	6	0.21
	Cellulose	5	0.17
	C/N	4	0.14
	Particle size	4	0.14
	Alkalinity	4	0.14
	Hemicellulose	3	0.10
	Pretreatment effect	3	0.10
	NH_3-N	3	0.10
	Lipid	3	0.10
	Protein	3	0.10
	Solid/water ratio	2	0.07
	Extractable content	2	0.07
	Detergent fiber	2	0.07
	Carbohydrates	2	0.07
**SP**	pH	4	1.00
	Ca^{2+}/PO_4^{3-}, Mg^{2+}/PO_4^{3-}, and NH_4^+/PO_4^{3-}	4	1.00
**AS**	Temperature	1	1.00
	pH	1	1.00
	NH_4^+-N load ratio	1	1.00
**Com**	C/N	1	1.00
	Extractable content	1	1.00
**HTT**	Temperature	3	1.00
	Time	3	1.00
	pH	1	0.33
	Solvent/feedstock ratio	1	0.33

Tech	Input variables	f	Rf
**Com**	C/N	3	1.00
	pH	3	1.00
	Electrical conductivity	3	1.00
	Temperature	2	0.67
	Feedstock quantity	2	0.67
	Time	1	0.33
	Moisture content	1	0.33
	Enzyme	1	0.33
	Dry matter	1	0.33
	NH_4^+/NO_3^-	1	0.33
**Pyr**	Temperature	54	1.00
	Time	29	0.54
	Heating rate	23	0.43
	Particle size	22	0.41
	Flow rate of N_2	15	0.28
	C-H-O-N content	9	0.17
	Ash content	8	0.15
	Moisture content	8	0.15
	Volatile matter	7	0.13
	Feedstock quantity	7	0.13
	Fixed carbon	5	0.09
	Loading rate	5	0.09
	Microwave power	4	0.07
	Lignin content	4	0.07
	Feedstock type	3	0.06
	Heating source	3	0.06
	Cellulose content	3	0.06
	Hemicellulose content	3	0.06
	Pressure	2	0.04
	Catalyst effect	1	0.02

Tech	Input variables	f	Rf
**Gas**	Temperature	37	1.00
	Equivalence ratio	19	0.51
	C-H-O-N content	19	0.51
	Steam or CaO/Feedstock	16	0.43
	Ash content	15	0.41
	Moisture content	14	0.38
	Time	7	0.19
	Fuel or air flow rate	5	0.14
	Volatile matter	5	0.14
	Blending ratio	4	0.11
	Particle size	4	0.11
	Pressure	4	0.11
	Fixed carbon	4	0.11
	Solid content	3	0.08
	Catalyst effect	3	0.08
	Feedstock type	2	0.05
	Feedstock concentration	2	0.05
	Loading rate	1	0.03
**HTT**	Temperature	30	1.00
	Time	29	0.97
	C-H-O-N content	11	0.37
	Feedstock/water	11	0.37
	Ash content	9	0.30
	Volatile matter	7	0.23
	Fixed carbon	6	0.20
	Catalyst effect	5	0.17
	Moisture content	4	0.13
	Loading rate	3	0.10
	Feedstock quantity	3	0.10
	Solid content	3	0.10
	Pressure	2	0.07
	Heating rate	2	0.07
	Lipid	2	0.07
	Protein	2	0.07
	Carbohydrates	2	0.07
	Lignin, Cellulose, and Hemicellulose content	1	0.03

Note: AD = anaerobic digestion, SP = struvite precipitation, AS = ammonia stripping, Com = composting, HTT = hydrothermal treatment, and Pyr = pyrolysis.

Table S9. Percentage (%) of different data-driven modeling applications in resource recovery and carbon capture literature.

**Technology**	**% of total applications**	**% of applications with respect to corresponding technology**									
		**Statistical methods**			**Machine learning methods**						
		**MLR**	**PLSR**	**MPR**	**ANN**	**ANFIS**	**SVM**	**DT**	**RF**	**XGBoost**	**GP**
AD	27.7	9.3	1.3	38.7	37.3	4.0	2.7	-	2.7	4.0	-
Com	3.0	37.5	12.5	25.0	25.0	-	-	-	-	-	-
Pyr	27.7	8.0	-	49.3	16.0	2.7	9.3	1.3	10.7	2.7	-
Gas	20.3	3.6	-	32.7	36.4	-	9.1	5.5	5.5	5.5	1.8
HTT	17.7	14.6	-	35.4	8.3	-	8.3	8.3	18.8	6.3	-
SP of liquid digestate	1.5	-	-	100.0	-	-	-	-	-	-	-
AS of liquid digestate	0.7	50.0	-	50.0	-	-	-	-	-	-	-
Com of solid digestate	0.4	-	100.0	-	-	-	-	-	-	-	-
HTT of solid digestate	1.1	66.7	-	33.3	-	-	-	-	-	-	-

Note: AD = anaerobic digestion, Com = composting, Pyr = pyrolysis, Gas = gasification, HTT = hydrothermal treatment, SP = struvite precipitation, AS = ammonia stripping, MLR = multiple linear regression, PLSR = partial least squares regression, MPR = multiple polynomial regression, ANN = artificial neural network, ANFIS = adaptive neuro fuzzy inference system, SVM = support vector machine, DT = decision tree, RF = random forest, XGBoost = extreme gradient boosting, GP = Gaussian process, and '-' = no applications.

Table S10. Percentage (%) of different environmental and economic analysis applications in resource recovery and carbon capture literature.

Technology	% of total applications	% of applications with respect to corresponding technology															
		Life cycle assessment methods										Techno-economic analysis methods					
		CML	IPCC	Eco-Indicator	EDIP	ReCiPe	TRACI	ILCD	Impact 2002+	GREET	Other	NPV	IRR	MSP	PBP	LCOE	ROI
AD	43.1	24.9	15.9	3.6	4.3	18.8	2.9	5.4	6.1	-	4.3	5.8	3.6	0.4	2.2	0.7	1.1
Com	7.6	28.6	10.2	2.0	14.3	16.3	8.2	4.1	-	-	8.2	6.1	-	-	-	-	2.0
Pyr	14.6	13.7	20.0	2.1	1.1	9.5	5.3	-	3.2	11.6	1.1	10.5	5.3	9.5	2.1	2.1	2.1
Gas	12.6	21.4	17.9	3.6	4.8	10.7	8.3	1.2	13.1	1.2	3.6	6.0	3.6	1.2	-	-	-
HTT	5.6	8.1	8.1	-	-	16.2	-	10.8	2.7	10.8	-	10.8	-	18.9	-	5.4	5.4
AD + SP	2.8	27.8	11.1	-	-	27.8	11.1	5.6	5.6	-	5.6	5.6	-	-	-	-	-
AD + AS	1.4	11.1	22.2	-	-	33.3	11.1	11.1	-	-	-	11.1	-	-	-	-	-
AD + SBR	0.3	50.0	50.0	-	-	-	-	-	-	-	-	-	-	-	-	-	-
AD + Com	4.7	18.8	9.4	3.1	-	25.0	3.1	6.3	-	-	3.1	9.4	6.3	-	6.3	3.1	-
AD + Pyr	2.2	14.3	21.4	-	-	28.6	7.1	-	-	-	14.3	7.1	7.1	-	-	-	-
AD + Gas	1.6	15.4	7.7	-	-	7.7	-	-	-	-	-	15.4	15.4	-	15.4	-	-
AD + HTT	0.5	-	25.0	-	-	-	-	-	-	-	-	25.0	25.0	-	-	-	-
AD + Gas + Other	0.3	-	-	-	-	-	-	-	-	-	-	50.0	-	-	50.0	-	-
AD + SP + CW	0.5	33.3	66.7	-	-	-	-	-	-	-	-	-	-	-	-	-	-
AD + AS + CW	0.5	33.3	66.7	-	-	-	-	-	-	-	-	-	-	-	-	-	-
AD + SP + Lag	0.2	-	-	-	-	100.0	-	-	-	-	-	-	-	-	-	-	-
AD + Com + Lag	0.2	100.0	-	-	-	-	-	-	-	-	-	-	-	-	-	-	-
AD + (SP + Com) + Other	0.2	-	-	-	-	100.0	-	-	-	-	-	-	-	-	-	-	-
AD + (AS + Com) + Other	0.2	-	-	-	-	100.0	-	-	-	-	-	-	-	-	-	-	-
AD + (AS + Pyr) + Other	0.3	-	-	-	-	50.0	-	-	-	-	-	50.0	-	-	-	-	-
AD + (AS + HTT) + Other	0.3	-	-	-	-	50.0	-	-	-	-	-	50.0	-	-	-	-	-

Note: AD = anaerobic digestion, Com = composting, Pyr = pyrolysis, Gas = gasification, HTT = hydrothermal treatment, SP = struvite precipitation, AS = ammonia stripping, SBR = sequencing batch reactor, CW = constructed wetlands, Lag = lagoon, Other = SBR, reverse osmosis, and tertiary treatment, and '-' = no applications.

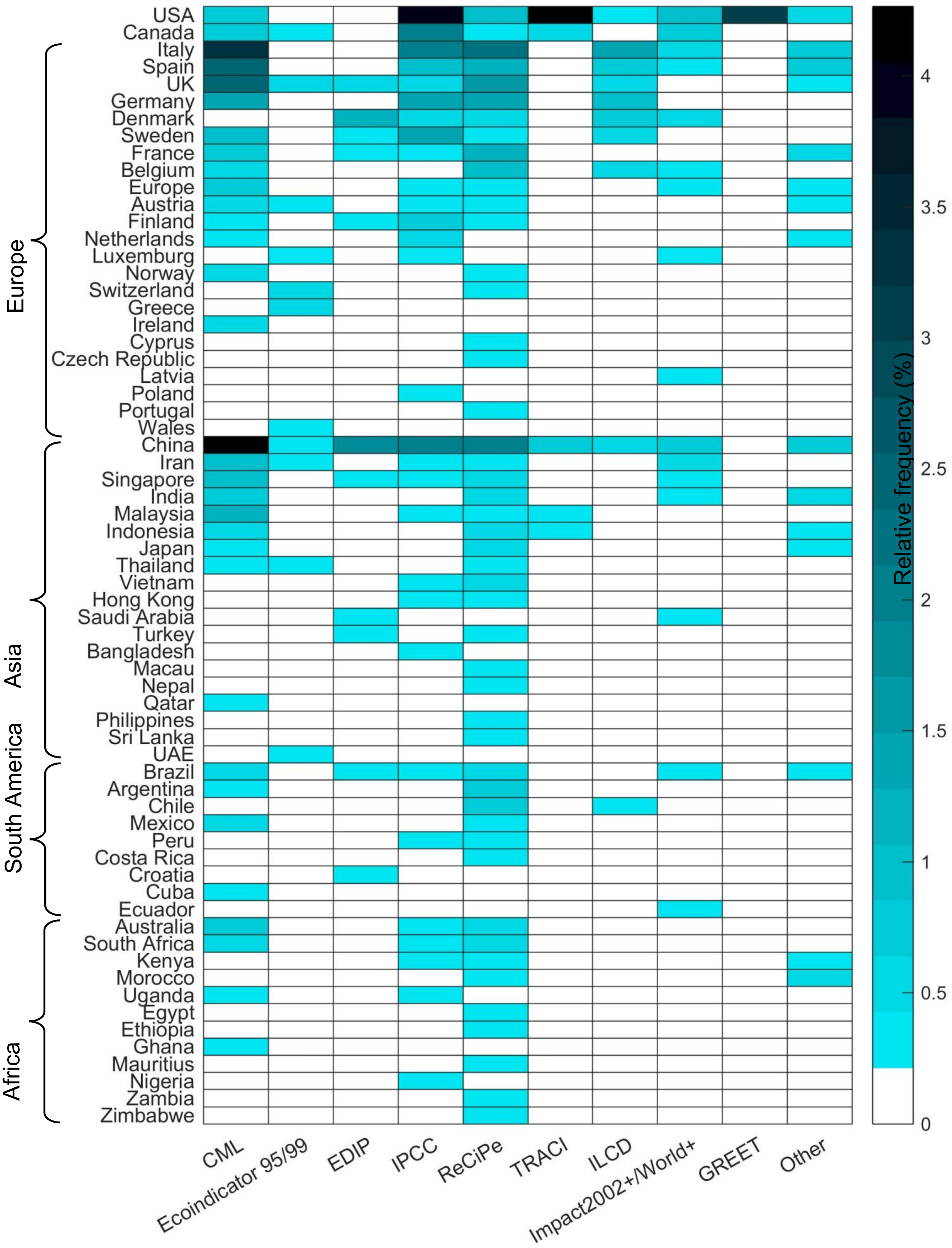

Figure S1. Number of applications of the various life cycle assessment methods with respect to the different countries around the world in resource recovery and carbon capture from organic waste literature.
